# Structural modification of fentanyls for their retrospective identification by gas chromatographic analysis using chloroformate chemistry

**DOI:** 10.1038/s41598-021-01896-x

**Published:** 2021-11-18

**Authors:** Carlos A. Valdez, Roald N. Leif, Robert D. Sanner, Todd H. Corzett, Mark L. Dreyer, Katelyn E. Mason

**Affiliations:** 1 Physical and Life Sciences Directorate, Lawrence Livermore National Laboratory, Livermore, CA 94550 USA; 2 Nuclear and Chemical Sciences Division, Lawrence Livermore National Laboratory, Livermore, CA 94550 USA; 3 Biosciences and Biotechnology Division, Lawrence Livermore National Laboratory, Livermore, CA 94550 USA; 4 Forensic Science Center, Lawrence Livermore National Laboratory, Livermore, CA 94550 USA

**Keywords:** Analytical chemistry, Organic chemistry

## Abstract

The one-step breakdown and derivatization of a panel of nine fentanyls to yield uniquely tagged products that can be detected by Electron Ionization Gas Chromatography-Mass Spectrometry (EI-GC-MS) is presented. The method involves the treatment of the synthetic opioids with 2,2,2-trichloroethoxycarbonyl chloride (TrocCl) at 60 °C for 3 h in dichloromethane and furnishes two products from one fentanyl molecule that can be used to retrospectively identify the original opioid. Parameters that were studied and fully optimized for the method included temperature, solvent, nature of scavenging base and reaction time. One of the two resulting products from the reaction bears the trichloroethoxycarbonyl (Troc) tag attached to the norfentanyl portion of the original opioid and greatly aids in the opioid detection and identification process. The methodology has been applied to the chemical modification of a panel of nine fentanyls and in all cases the molecular ion peak for the Troc-norfentanyl product bearing the distinctive trichloroethyl isotopic signature can be clearly observed. The method’s LLOD was determined to be 10 ng/mL while its LLOQ was found to be 20 ng/mL. This methodology represents the first application of chloroformates in the chemical modification of this class of synthetic opioids that are notoriously inert to common derivatization strategies available for GC–MS analysis.

## Introduction

**Figure 1:**
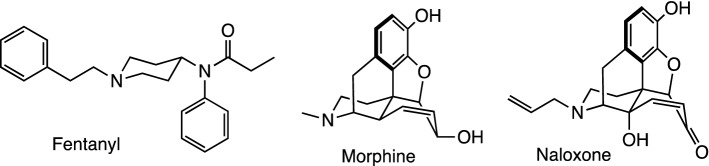
Structures of fentanyl, morphine and the most common antidote employed in cases of fentanyl poisoning, naloxone.

Since its discovery in the laboratory of Paul Janssen in 1960^1^, fentanyl (Fig. 1) has become one of the gold standards in the medical field for use as a safe anesthetic during perioperative surgical procedures and in the management of pain in various disease states^2,3^. The medical practitioners’ preference for fentanyl over morphine (Fig. 1) stems from its faster onset time, stronger potency and lower risk for acute heart and respiratory failure in the patient^4,5^. Unfortunately, the recognized beneficial impact of this synthetic opioid has been opaqued by its involvement in numerous deaths stemming from its illicit consumption^6,7^. To further augment their notoriety, fentanyls have been become part of a specialized group of chemical warfare agents known as incapacitating agents^8^. Their employment in the Dubrovka Theater siege in Moscow epitomized their power to cause mass casualties^9,10^. Due to their emergence as a real threat to public health as proven by the outbreak of numerous pandemic cases worldwide and the alarming rate of production by clandestine laboratories using published protocols^11–13^, many efforts are now being directed at counteracting their effects^14,15^ with drugs like naloxone (Fig. 1) as well as developing more efficient detection methods using Gas Chromatography-Mass Spectrometry (GC–MS)^16,17^ and Liquid Chromatography-Mass Spectrometry (LC–MS)^18^ instrumentation.

In the field of analytical chemistry, fentanyl detection has mostly been accomplished by LC–MS methods that features detection limits for the opioid down to the picogram level^19^. Analysis and detection by LC–MS means of these opioids is further aided by their UV chromophores as well as their basic nitrogen that in the salt form exists in a protonated state increasing the drugs’ solubility in the aqueous gradients used. In contrast, GC–MS has not encountered the same level of success in detecting fentanyl at such low levels and this can be attributed to several factors. One that stands out is that many of the fentanyls encountered in biological matrices or in the environmental setting are in their salt form (either hydrochloride or citrate salts) that require a basic sample preparation step that ultimately converts it to the free base for GC detection^17,20,21^. Another is the chemical inertness of fentanyl itself and analogs that effectively hinders the use of derivatizing agents (e.g. silylation, methylation) for converting them into species with enhanced chromatographic profiles for GC–MS analysis. Thus, the only effective reaction of fentanyl itself, and simple analogs thereof, is the one involving its tertiary piperidinyl nitrogen with an acid to form a salt, as there are no other reactive functionalities in the molecule to exploit from a derivatization standpoint. Naturally, more complex fentanyls, where the presence of hydroxyl and carboxyester groups, offer more opportunities for chemical modification via established derivatization techniques.

Having the ability to identify these synthetic opioids in a sample by GC–MS with high degree of confidence would be an invaluable tool in the analytical field. GC–MS is an analytical technique that offers several advantages over other techniques, including LC–MS, as a means of analyzing this class of compounds. GC–MS instrumentation can be found virtually in every analytical chemistry laboratory, largely due to its much lower cost for purchase, maintenance and operation. In addition, the inclusion of mass spectral data in collections like the NIST and OCAD libraries make the initial identification of an unknown material a fairly simple task at an early stage during sample analysis^22–24^. Therefore, providing the analyst with not only standards for direct comparison but a derivatization reaction that can unambiguously identify the opioid in the mixture can be of great complementary value. The analysis of fentanyl and structurally-related congeners by GC–MS can be a daunting task if a sample contains a new analog that has not been previously encountered and for which no library analysis and match can be obtained. In these instances, a derivatization reaction often solves the problem of unambiguously identifying an unknown substance while still relying on a single analytical technique like GC–MS. To date no direct derivatization protocol for fentanyls exists and thus no alternate way of analyzing these by GC–MS can be accomplished. In this work, we introduce a protocol involving the chemical modification of fentanyls that yields two products possessing vestigial features of the starting fentanyl that can be pieced together afterwards to retrospectively and unambiguously identify the original opioid.

## Methods

### Materials

All chemicals were purchased from commercial suppliers and used as received. 2,2,2-trichloroethoxycarbonyl chloride (TrocCl), triethylamine (TEA), diisopropylethylamine (DIPEA), tetramethylpiperidine (TMP), acetonitrile (ACN), acetone, ethyl acetate and dichloromethane were purchased from Sigma-Aldrich (St. Louis, MO.). Sodium bicarbonate and anhydrous sodium sulfate were purchased from Acros Organics (Westchester, PA.). Deuterated chloroform (CDCl_3_) and sodium carbonate were purchased from Alfa Aesar (Ward Hill, MA). Acrodisc PTFE syringe filters (0.45 μm) were purchased from Pall laboratories (Port Washington, NY.). Autosampler vials and glass inserts were purchased from Agilent Technologies (Santa Clara, CA.). All the fentanyls described in this paper were synthesized using published protocols and their NMR spectra matched the published ones. For isobutyrylfentanyl and valeroylfentanyl, their syntheses were accomplished by a modification of the synthesis for fentanyl^11^ and their associated NMR spectra are included in the Supporting Information (Pages S11-S13) section. All fentanyls and the Troc-norfentanyl standard were purified by flash column chromatography using a Biotage Isolera purification system using SNAP KP-Si silica gel column cartridges.

### EI-GC-MS analysis method

A 6890 Agilent GC with 5975 MS detector equipped with a split/splitless injector was used for the analysis as previously described^25–29^. The GC column used for the analysis was an Agilent HP- 5 ms UI capillary column (30 m × 0.25 mm id × 0.25 μm film thickness). Ultra-high purity helium, at 0.8 mL/min, served as the carrier gas. The inlet was operated in pulsed splitless mode (25 psi for 1 min, followed by a 50 mL/min purge flow), with the injector temperature set at 250 °C and the injection volume was 1 μL. The oven temperature program was as follows: 40 °C, held for 3 min, increased at 8 °C/min to 300 °C, held for 3 min. The MS ion source and quadrupole temperatures were 230 °C and 150 °C, respectively. Electron ionization (EI) was used with an ionization energy of 70 eV. The MS was operated to scan from *m/z* 29 to 600 in 0.4 s with a solvent delay of 3.5 min.

### GC–MS analysis method for LLOD and LLOQ

Established methods, for both GC–MS and LC–MS, exist for the intact analysis of fentanyl and related opioids and these guided our LLOD and LLOQ values determination using a pure sample of Troc-norfentanyl synthesized in our lab for this purpose^30–32^. Sample analyses were performed on an Agilent 6890 GC coupled to an Agilent 5975c MS detector. The GC column used for the analysis was an Agilent DB-17ht capillary column (30 m × 0.25 mm id × 0.15 μm film thickness). Ultra-high purity helium served as the carrier gas. The inlet was operated in constant pressure mode (25 psi, with inlet purge at 1 min at 50 mL/min purge flow), with the injector temperature set at 285 °C and injection volumes of 1 μL. The oven temperature program was as follows: 140 °C, held for 1 min, increased at 25 °C/min to 325 °C, held for 1.6 min. The MS ion source and quadrupole temperatures were 230 and 150 °C, respectively. Electron ionization was used with an ionization energy of 70 eV. The MS was operated to SIM mode (m/z 93, 132, 149, and 259), with a solvent delay of 5 min.

### Nuclear magnetic resonance

Spectra were obtained using a Bruker Avance III 600 MHz instrument equipped with a Bruker QNP 5 mm cryoprobe (Bruker Biospin, Billerica, MA) at 30.0 ± 0.1 °C. NMR data is reported as follows: chemical shift (δ) (parts per million, ppm); multiplicity: m (multiplet), d (doublet), t (triplet), q (quartet), app t (apparent triplet), tt (triplet of triplets), qd (quartet of doublets), quin (quintet), sep (septet); coupling constants (*J*) are given in Hertz (Hz). ^1^H NMR (600 MHz) chemical shifts are calibrated with respect to residual DMSO-_d5_ in DMSO-_d6_ centered at 2.50 ppm, whereas for ^13^C NMR (151 MHz), the center peak for DMSO-_d6_, centered at 39.52 ppm, was used for the spectral calibration. For acquisitions in CDCl_3_, chemical shifts are calibrated with respect to residual chloroform in CDCl_3_ centered at 7.26 ppm, whereas for ^13^C NMR the center peak for CDCl_3_, centered at 77.0 ppm, was used for the spectral calibration. ^13^C-DEPT-135 NMR was used to identify the nature (i.e. 1°, 2°, 3° or quaternary) of the carbon atoms in the synthesized targets.

### Chemical synthesis

#### Isobutyrylfentanyl

1-phenethyl-*N*-phenylpiperidin-4-amine (4-ANPP, 100 mg, 0.36 mmol)^11^ was dissolved in anhydrous dichloromethane (15 mL) in a 50 mL round bottom flask equipped with a stir bar. The solution was cooled with an ice bath (~ 4–5 °C) and treated sequentially with triethylamine (106 μL, 0.79 mmol, 2.2 equiv.) and isobutyryl chloride (79 μL, 0.79 mmol) via pipette dropwise. The ice bath was removed and the reaction stirred at ambient temperature overnight. The following day, the pale yellow suspension was transferred to a 250 mL separatory funnel and partitioned with deionized water (50 mL). The organic phase was extracted with brine (NaCl/H_2_O, 50 mL), dried over anhydrous sodium sulfate and evaporated *in vacuo* at 40 °C to give a dark yellow oily mixture. The mixture was purified by flash column chromatography (0 → 10% MeOH/DCM) to furnish isobutyrylfentanyl as a light yellow solid (93 mg, 74%). ^1^H NMR (600 MHz, DMSO-_d6_) δ 7.48–7.46 (m, 2H), 7.44–7.42 (m, 1H), 7.24–7.19 (m, 4H), 7.15–7.13 (m, 3H), 4.40 (tt, *J* = 12.1, 3.8 1H), 2.90 (d, *J* = 11.6, 2H), 2.63 (app t, *J* = 7.4, 2H), 2.48 (app t, *J* = 8.2, 2H), 2.11 (sep, *J* = 6.7, 1H), 1.99 (t, *J* = 11.8, 2H), 1.66 (d, *J* = 11.6, 2H), 1.16 (qd, *J* = 12.1, 3.7 2H), 0.90 (d, *J* = 6.7, 6H); ^13^C NMR (151 MHz, DMSO-_d6_) δ 175.7 (C = O), 141.1, 139.4, 130.8, 129.9, 129.1, 128.9, 128.8, 126.4, 60.2, 53.1, 52.4 (C-H), 33.6, 31.8 (C-H, *i*Bu), 30.7, 20.1 (2 × CH_3_); HRMS (CI) *m/z* calcd for C_23_H_31_N_2_O [M + H]^+^: 351.2436; found 351.2413.

#### Valeroylfentanyl

The same protocol above was followed for the synthesis of valeroylfentanyl with the only difference that valeroyl chloride (79 μL, 0.79 mmol) was used instead of isobutyryl chloride. Valeroylfentanyl was obtained after purification as a yellow solid (85 mg, 65%). ^1^H NMR (600 MHz, DMSO-_d6_) δ 7.46 (app t, *J* = 7.6, 2H), 7.41 (app t, *J* = 7.3, 1H), 7.24–7.20 (m, 2H), 7.18 (d, *J* = 7.3, 2H), 7.15–7.13 (m, 3H), 4.44–4.40 (m, 1H), 2.90 (d, *J* = 11.6, 2H), 2.64–2.61 (m, 2H), 2.44–2.41 (m, 2H), 1.99 (t, *J* = 10.5, 3H), 1.81 (t, *J* = 7.4, 2H), 1.68–1.66 (m, 2H), 1.39 (quin, *J* = 7.4, 2H), 1.21–1.14 (m, 2H), 1.12–1.09 (m, 2H), 0.72 (t, *J* = 7.3, 3H); ^13^C NMR (151 MHz, DMSO-_d6_) δ 171.6 (C = O), 141.1, 139.5, 130.9, 129.9, 129.2, 129.1, 128.8, 126.4, 60.2, 53.1, 52.4 (C−H), 34.6, 33.6, 30.8, 27.6, 24.6, 22.3 (CH_3_); HRMS (CI) *m/z* calcd for C_24_H_33_N_2_O [M + H]^+^: 365.2593; found 365.2584.

#### General method description with optimized conditions

In a typical protocol involving 3 μmol of the fentanyl in DCM (200 μL) is treated with an inorganic base (300 μmol) in an autosampler vial equipped with a micro-stir bar. To this solution, TrocCl (300 μmol) was added via pipette, the vial capped and heated to 60 °C in an aluminum heating block for 3 h with vigorous stirring. After 3 h, the vial was removed from the heating block and the mixture allowed to cool to ambient temperature. An aliquot of the reaction mixture (100 μL) was removed via pipette and transferred to another autosampler vial equipped with a glass insert for GC analysis (injection volume: 1 μL).

## Results and discussion

**Figure 2:**
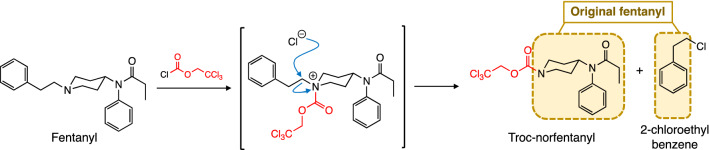
Outline of derivatization strategy described in this work. Treatment of a fentanyl with TrocCl results in an intermediate that undergoes a chloride-mediated dealkylation to furnish two products: Troc-norfentanyl and 2-(chloroethyl)benzene. The dashed boxed sections in both products can be retrospectively joined to reveal the identity of the original fentanyl.

Structurally, fentanyl itself is an unreactive molecule, lacking functionalities that can be exploited for chemical derivatization to further enhancing its detection and identification by GC–MS. However, this is not the case for other members of this family like carfentanil and remifentanil with reactive ester sites. Almost fifty years ago, a report from the Portoghese group at the University of Minnesota described their studies on the reaction of various morphine-related compounds with chloroformate esters^33^. In these studies the authors found that treatment of morphine with phenyl chloroformate resulted in its N-demethylation with concomitant replacement of this methyl group by the alkyl portion of the chloroformate employed leading to the formation of a carbamate analog of the alkaloid. These studies were largely based on the now century-old report by Gadamer and Knoch in 1921 on the demethylation of tertiary amine in bullcapnine^34^. Since that report, work by other groups expanded the use of other various chloroformates during organic synthesis manipulations^35–38^. As part of our program at the Forensic Science center (FSC) is deeply involved with the development of analytical methods for the analysis of these opioids by GC–MS means, we found this report very interesting and noticed its potential application in the field of fentanyl chemistry. Given that fentanyl and analogs thereof possess an N-alkyl group off the piperidine ring nitrogen showing a similar degree of steric hindrance as the methyl group, we anticipated that fentanyl should undergo the dealkylation/carbamate formation process in similar fashion to morphine when reacting with a chloroformate. In principle, this reaction would lead to the formation of two products that will have elements belonging to the original fentanyl they originated from (Fig. 2).

In analogous fashion to the morphine nucleus, we hypothesized that treatment of a fentanyl with trichloroethoxyethyl chloroformate (Troc-Cl) would result in a quaternary intermediate that undergoes a nucleophilic attack by the generated chloride ion at the phenylethyl side chain to produce two products: Troc-norfentanyl and 2-(chloroethyl)benzene (Fig. 2). The two products generated in this process can be used to retrospectively confirm the identity of the starting fentanyl even if this one cannot be detected in its intact form in a given matrix (e.g. biological in nature). An important part of the conjectured process is that replacement of the phenylethyl side chain is the preferred route for the reaction over attack of the chlorine atom at the adjacent carbons forming the piperidine ring. However, steric hindrance provided by the axial protons in the cyclic piperidine ring favors attack of the Cl^−^ anion at the phenylethyl side chain. What follows below are our optimization studies on various components of the reaction. These optimization studies included a screening of bases (organic and inorganic) for the reaction and assessment of the temperature, solvent and time for the transformation.

### Assessment of scavenging base for the reaction

**Figure 3:**
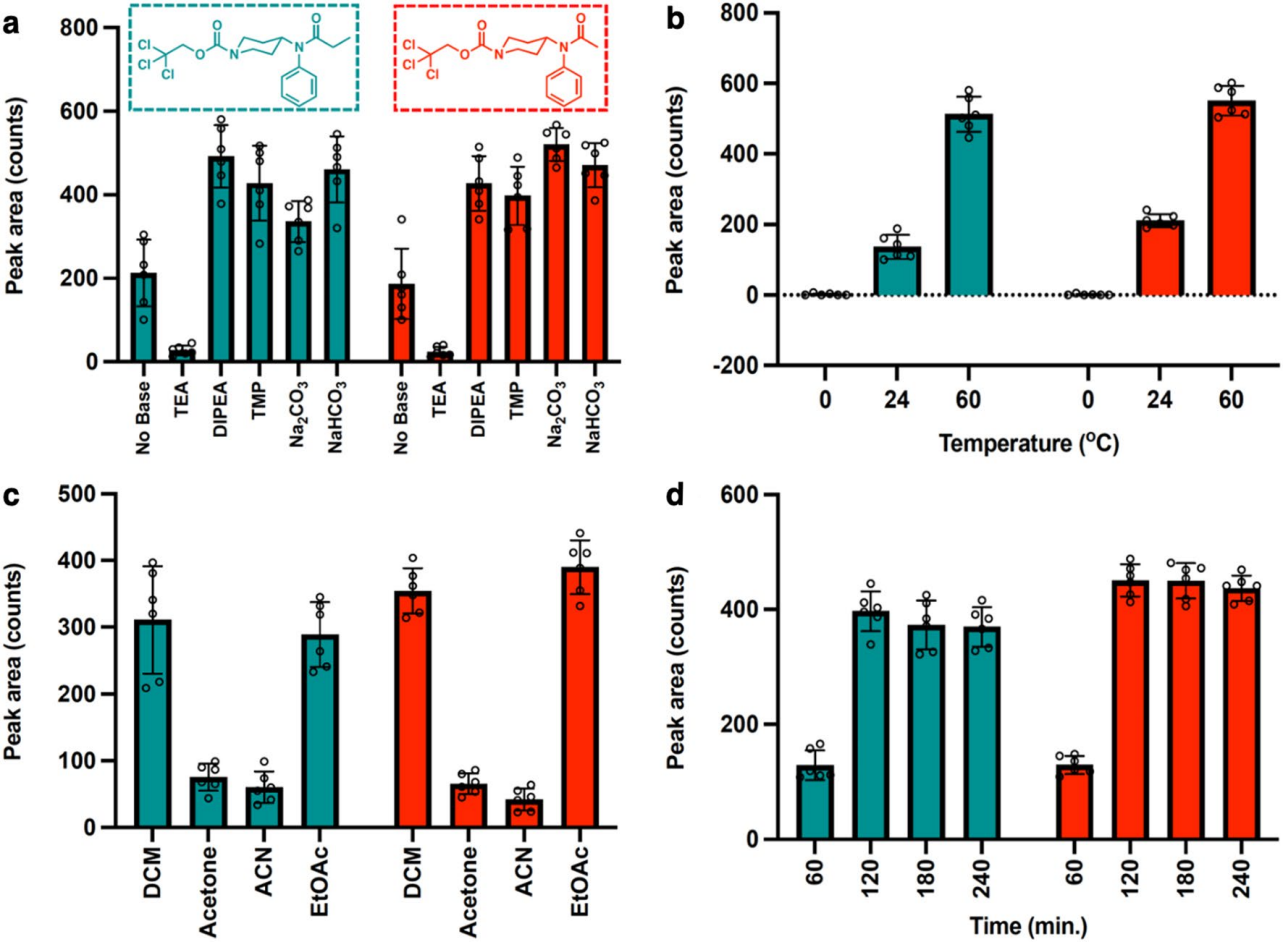
Optimization of reaction parameters for the reaction between fentanyl and acetylfentanyl with TrocCl (n = 6, ± standard deviation for each bar graph presented). (**a**) Effect of the nature of the base in the production of Troc-norfentanyl (teal bars) and Troc-nor-acetylfentanyl (red bars) at 60 °C at t = 3 h.; (**b**) effect of temperature on the reaction demonstrating the increase of the Troc-norfentanyl products as temperature is increased; (**c**) effect of solvent on the reaction showing its higher performance in DCM and ethyl acetate (EtOAc); (**d**) effect of time on the reaction.

Our initial studies involved the modification of fentanyl using the protocol. In general, trichloroethoxycarbonylation reactions are conducted over a range of temperatures and using scavenging bases that can be organic, nitrogenous species like triethylamine (TEA)^39^ or inorganic ones like sodium hydroxide. Interestingly, when triethylamine was initially screened for use in the reaction, the major product observed in the mixture was the one arising from the reaction between TEA and TrocCl, namely 2,2,2-trichloroethyl diethylcarbamate. As both components were used in excess relative to fentanyl, the dominant species in the mixture was 2,2,2-trichloroethyl diethylcarbamate, while the Troc-norfentanyl seemed to only marginally form. By analyzing the proposed mechanism for the reaction, we realized two things, one was that the generation of 2,2,2-trichloroethyl diethylcarbamate confidently pointed towards the success of the protocol with the opioid, and the other was that we can possibly use more hindered nitrogenous bases so as to avoid their reaction with TrocCl, and simply behave as proton sponges in the procedure. With this in mind, we decided to evaluate more hindered bases for this purpose such as diisopropylethylamine (DIPEA) and tetramethylpiperidine (TMP), with the added benefit that both are liquids at room temperature and miscible in DCM. Another set of bases explored were the inorganic ones and these included sodium bicarbonate, potassium bicarbonate and sodium hydroxide. Now, due to the solid nature of these inorganic bases and their insolubility in DCM, we expected for these reactions to be biphasic in nature and potentially not been able to scavenge the generated acid as efficiently as their organic counterparts. To our surprise, reaction mixtures involving the inorganic bases yielded clean GC–MS chromatograms mainly due to the insolubility of these bases in the DCM. In contrast, DIPEA and TMP provided large signals that dominated the GC chromatogram. Additionally, the reaction was also carried out in the absence of a base and although it did produce more of the Troc-norfentanyl product than when TEA was used, it was not as efficient as the cases where the hindered or inorganic bases were used. One possible explanation for the reduced performance in this case involves the reaction of the generated HCl in the first step with another fentanyl molecule effectively protonating it and making it unavailable to react with TrocCl. Figure 3a shows a bar graph that summarizes the formation of the products, Troc-norfentanyl (teal-colored bars) and Troc-acetylfentanyl (red-colored bars), as a function of the base used.

### Assessment of temperature for the reaction

Reactions between TrocCl and amines during protective group installations in organic synthesis are routinely carried out at ambient temperature and even at 4 °C (ice bath)^40,41^. In our case, we are not using the reagent to carry out a one-step protection but to efficiently break apart a molecule at its tertiary amine point of junction. In our specific case, we are not only relying on the initial carbamate formation but also on the subsequent displacement of the phenylethyl side chain by the chloride ion, and for this entire process we thought that heating might be a necessary component. To this end, we analyzed the formation of the Troc-norfentanyl products, originating from the reaction between TrocCl and fentanyl and acetylfentanyl separately, under three different temperatures: 4, 24 and 60 °C. The results are summarized on Fig. 3b, and it can be appreciated that increasing the temperature of the reaction leads to the formation of more Troc-norfentanyl (teal-colored bars) and Troc-noracetylfentanyl (red-colored bars) products.

### Solvent effects for the reaction

Following our assessment of temperature effects in the reaction, we turned to studying the effect of solvent polarity in the overall yield of the Troc-norfentanyl and Troc-noracetylfentanyl products. The results are presented in Fig. 3c and it can be concluded that product formation is heavily favored in solvents like DCM and ethyl acetate, while their formation is less favored in solvents like acetone and acetonitrile (ACN). Regarding these results one can invoke several factors at play such as the solvent’s polarity for which we can use dielectric constant values (ε) to create a conjecture. Based on this, one can argue that the reaction proceeds well in solvents with lower dielectric constants (ε) like DCM (ε = 8.9) and EtOAc (ε = 6.0) rather than ones featuring higher dielectric constants like acetone (ε = 20.7) and ACN (ε = 37.5). This actually favors nucleophilic attack by the chloride ion as there is no tight coordination of the solvent molecules to the Cl^−^ ion, even though stabilization of the ionic species might not be favored heavily in these solvents, however all this is overweighed by the fact that heating is a vital characteristic of this protocol.

### Optimizing reaction time and application of method to other fentanyls

Lastly, we assessed the accumulation of the Troc-norfentanyl and Troc-nor-acetylfentanyl products as a function of time. The selected time points that were chosen for EI-GC-MS analyses were 30, 60, 90, 120 and 240 min. The results are summarized in Fig. 3d and demonstrates that most of the product accumulates after two hours with this concentration becoming steady over the four-hour period that the reaction was monitored.

**Figure 4:**
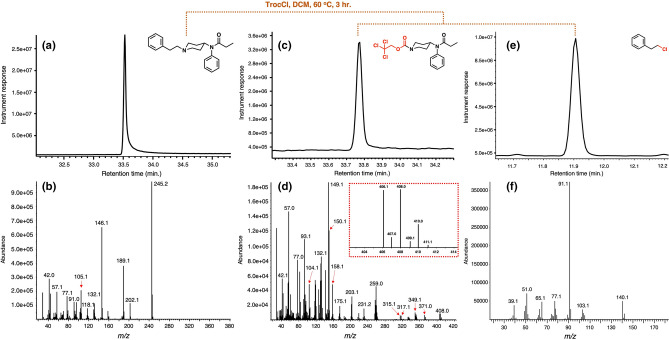
Trichloroethoxycarbonylation of fentanyl using optimized reaction conditions. (**a**) GC chromatogram of fentanyl (free base, rt = 33.5 min.; (**b**) mass spectrum of fentanyl; (**c**) GC chromatogram of first reaction product, Troc-norfentanyl (rt = 33.7 min.); (**d**) mass spectrum of Troc-norfentanyl showing isotopic pattern of its molecular ion peak in yellow inset; (**e**) GC chromatogram of second reaction product, 2-chloroethylbenzene (rt = 11.9 min.); (**f**) mass spectrum of 2-chloroethylbenzene.

To this end and after putting together the most optimized conditions for the reaction, we moved on to test the reaction’s performance with other members of this class of opioids. The results of the protocol against a panel of 9 fentanyls are summarized in Table 1. As it can be observed all the fentanyls reacted in similar fashion to provide two products, the chloride arising from the N-alkyl side chain excision during the process and the Troc-norfentanyl fragment, becoming unique markers for each fentanyl. The results in Table 1 also show that the method appears to be universal for this class of synthetic opioids providing the predicted Troc-norfentanyl product every time. An appealing part of the protocol is the production of a non-natural nor-fentanyl marker in each case (i.e. Troc-norfentanyl) that can be used, in conjunction with the N-alkyl chloride originating from the N-piperidinyl side chain of the fentanyl, to retrospectively identify the original opioid. Figure 4 shows the application of the optimized reaction conditions for the fentanyl:TrocCl system. One interesting observation is the fact that no molecular ion peak can be observed for the fentanyl (Fig. 4b) as well as for the other eight analogs studied in this work (See Supporting Information, Pages S3-S11). In contrast, molecular ion peaks for Troc-norfentanyl and 2-chloroethylbenzene can be clearly observed (Figs. 4d and f). The mass spectrum for 2-chloroethylbenzene can be found in the instrument’s internal library and can be used to identify the material with high degree of confidence (Match score: 920). The spectrum is quite simple with few diagnostic peaks (including the molecular ion peak with m/z = 140.1) and its base peak (m/z = 91.1) that can be used to identify the material by using the ion extraction mode in the instrument’s software. In contrast, Troc-norfentanyl features a more complex mass spectrum full of key peaks that are unique for this material (Fig. 4d). The molecular ion peak at m/z = 406.1, even though not one of the most intense peaks in the spectrum is still clearly visible and features the trademark isotopic pattern that arises from the three chlorine atoms in the Troc tag (Fig. 4d, inset). The mass spectrum for Troc-norfentanyl also features a peak at m/z = 371.0 [M–Cl]^+^ representing a loss of a chlorine and a peak at m/z = 349.1 [M–C_3_H_5_O]^+^ representing scission of the N-propanoyl moiety. The base peak with a m/z = 149.1 appears to originate from the cleavage of the C-N bond at the 4-position of the piperidine ring resulting in a species with the general formula [C_9_H_11_NO]^+^. The method’s lower limit of detection (LLOD) and lower limit of quantitation (LLOQ) were determined and the LLOD value was defined as the concentration of Troc-norfentanyl that provided a signal-to-noise value between 3 and 10 for 4 diagnostic ions (*m/z* = 93, 132, 149, and 259) while the LLOQ value was the concentration of Troc-norfentanyl that provided a signal-to-noise value > 10 for the 4 diagnostic ions (See Supporting Information, pages S21-S39). Both the LLOD and LLOQ values had acceptable precision and accuracy using percent coefficient of variation (%CV) < 20%. The method’s LLOD and LLOQ were found to be 10 ng/mL and 20 ng/mL and where determined using a pure sample of Troc-norfentanyl.

**Table 1:**
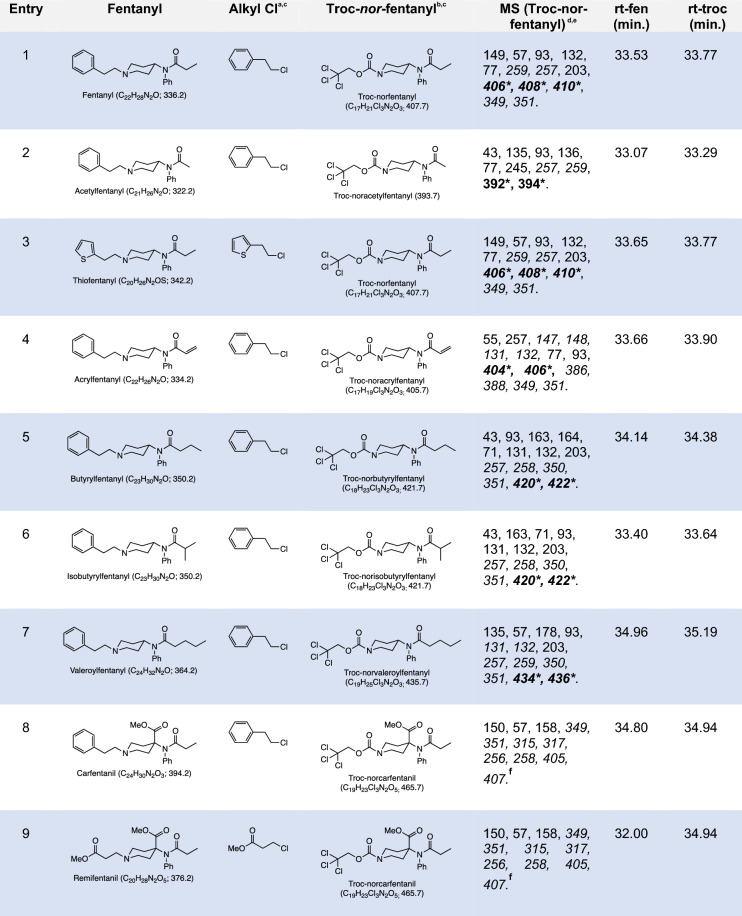
^a^Alkyl chloride from N-alkyl group after reaction; ^b^Troc-norfentanyl fragment after reaction; ^c^Product yields based on GC–MS; ^d^Mass fragments present in the Troc-norfentanyl product after reaction; ^e^Mass fragments in italics belong to chlorine-containing clusters, while italicized and bold-faced fragments bearing an asterisk indicate they are part of the molecular ion peak; ^f^ Molecular ion peaks visible upon closer inspection of the spectrum but are in very low abundance.

## Conclusions

We have demonstrated in this work that the reaction between the synthetic opioid fentanyl and the chloroformate 2,2,2-trichloroethoxycarbonyl chloride (TrocCl) yields two products of predictable structures. The first one is 2-phenylethyl chloride arising from fentanyl’s N-side chain while the other one has been identified as Troc-*nor*-fentanyl. After optimizing the conditions for the reaction, the method was tested on a panel of nine fentanyls to yield uniquely tagged products that can be detected by EI-GC-MS. The methodology described herein represents the first application of the use of chloroformate chemistry to chemically modify this class of synthetic opioids that are notoriously inert to derivatizing agents available for GC–MS analysis. Current work is focusing on the applicability of the protocol for effectively modifying and detecting these opioids in biological sample matrices.

## Supplementary Information

Supplementary Information.
