# Supplementary material for: Structural modification of fentanyls for their retrospective identification by gas chromatographic analysis using chloroformate chemistry
Source: Sci Rep. 2021 Nov 18;11:22489. doi: 10.1038/s41598-021-01896-x (PMC8602620; doi:10.1038/s41598-021-01896-x)
Supplement: Supplementary file 1 — Supplementary Information. [file 41598_2021_1896_MOESM1_ESM.pdf]

-Supporting Information-

"Structural Modification of Synthetic Opioids for their Retrospective Identification by Gas Chromatographic Analysis Using Chloroformate Chemistry"

-Table of Contents-

Content	Page
EI-GC-MS analysis method for fentanyls	S2
GC-MS Analysis Method for LLOD and LLOQ determination	S2
Figure S1. GC-MS of fentanyl	S3
Figure S2. GC-MS of Troc-norfentanyl	S3
Figure S3. GC-MS of acetylfentanyl	S4
Figure S4. GC-MS of Troc-noracetylfentanyl	S4
Figure S5. GC-MS of thiofentanyl	S5
Figure S6. GC-MS of Troc-northiofentanyl	S5
Figure S7. GC-MS of butyrylfentanyl	S6
Figure S8. GC-MS of Troc-norbutyrylfentanyl	S6
Figure S9. GC-MS of isobutyrylfentanyl	S7
Figure S10. GC-MS of Troc-norisobutyrylfentanyl	S7
Figure S11. GC-MS of valeroylfentanyl	S8
Figure S12. GC-MS of Troc-norvaleroylfentanyl	S8
Figure S13. GC-MS of carfentanil	S9
Figure S14. GC-MS of Troc-norcarfentanil	S9
Figure S15. GC-MS of remifentanil	S10
Figure S16. GC-MS of Troc-norremifentanil	S10
Figure S17. GC-MS of acryloylfentanyl	S11
Figure S18. GC-MS of Troc-noracryloylfentanyl	S11
Nuclear Magnetic Resonance (NMR) acquisition method	S12
Figure S19. ^{1}H NMR spectrum of valeroylfentanyl	S12
Figure S20. ^{13}C NMR spectrum of valeroylfentanyl	S13
Figure S21. ^{13}C-DEPT-135 NMR spectrum of valeroylfentanyl	S13
Figure S22. ^{1}H NMR spectrum of isobutyrylfentanyl	S14
Figure S23. ^{13}C NMR spectrum of isobutyrylfentanyl	S14
Figure S24. ^{13}C-DEPT-135 NMR spectrum of isobutyrylfentanyl	S15
Scheme S1. Synthesis of Troc-norfentanyl	S16
Figure S25. ^{1}H NMR spectrum of compound **2**	S18
Figure S26. ^{13}C NMR spectrum of compound **2**	S18
Figure S27. ^{1}H NMR spectrum of compound **3**	S19
Figure S28. ^{13}C NMR spectrum of compound **3**	S19
Figure S29. ^{1}H NMR spectrum of Troc-norfentanyl (**4**)	S20
Figure S30. ^{13}C NMR spectrum of Troc-norfentanyl (**4**)	S20
Figure S31. ^{13}C-DEPT-135 NMR spectrum of Troc-norfentanyl (**4**)	S21
GCMS data (raw) for LLOD/LLOQ determination	S22

EI-GC-MS Analysis Method. A 6890 Agilent GC with 5975 MS detector equipped with a split/splitless injector was used for the analysis as previously described. The GC column used for the analysis was an Agilent HP- 5ms UI capillary column (30 m × 0.25 mm id × 0.25 μm film thickness). Ultra-high purity helium, at 0.8 mL/min, served as the carrier gas. The inlet was operated in pulsed splitless mode (25 psi for 1 minute, followed by a 50 mL/min purge flow), with the injector temperature set at 250 °C and the injection volume was 1 μL. The oven temperature program was as follows: 40 °C, held for 3 min, increased at 8 °C/min to 300 °C, held for 3 min. The MS ion source and quadrupole temperatures were 230 °C and 150 °C, respectively. Electron ionization (EI) was used with an ionization energy of 70 eV. The MS was operated to scan from *m/z* 29 to 600 in 0.4 sec with a solvent delay of 3.5 min.

GC-MS Analysis Method for LLOD and LLOQ. Established methods, for both GC-MS and LC-MS, exist for the intact analysis of fentanyl and related opioids and these guided our LLOD and LLOQ values determination using a pure sample of Troc-norfentanyl synthesized in our lab for this purpose[30-32]. Sample analyses were performed on an Agilent 6890 GC coupled to an Agilent 5975c MS detector. The GC column used for the analysis was an Agilent DB-17ht capillary column (30 m × 0.25 mm id × 0.15 μm film thickness). Ultra-high purity helium served as the carrier gas. The inlet was operated in constant pressure mode (25 psi, with inlet purge at 1 minute at 50 mL/min purge flow), with the injector temperature set at 285 °C and injection volumes of 1 μL. The oven temperature program was as follows: 140 °C, held for 1 min, increased at 25 °C/min to 325 °C, held for 1.6 min. The MS ion source and quadrupole temperatures were 230 and 150 °C, respectively. Electron ionization was used with an ionization energy of 70 eV. The MS was operated to SIM mode (m/z 93, 132, 149, and 259), with a solvent delay of 5 min.

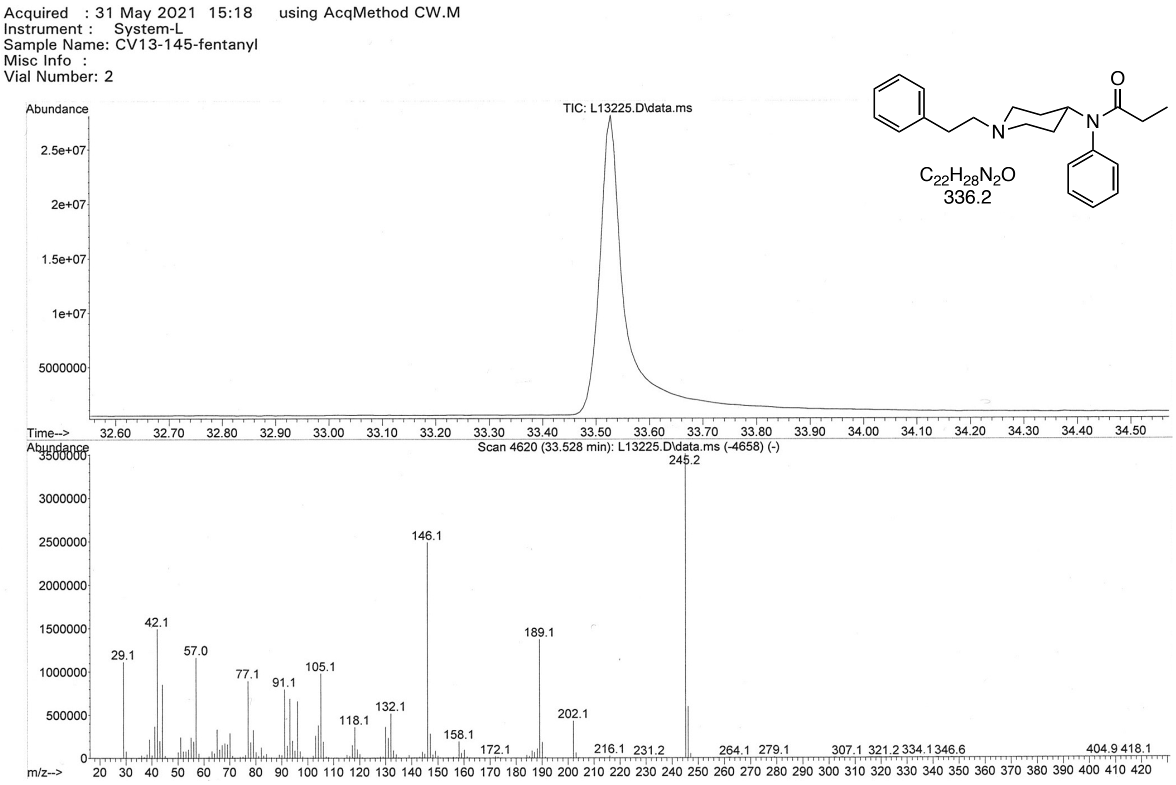

Figure S1. GC-MS chromatogram and MS for fentanyl.

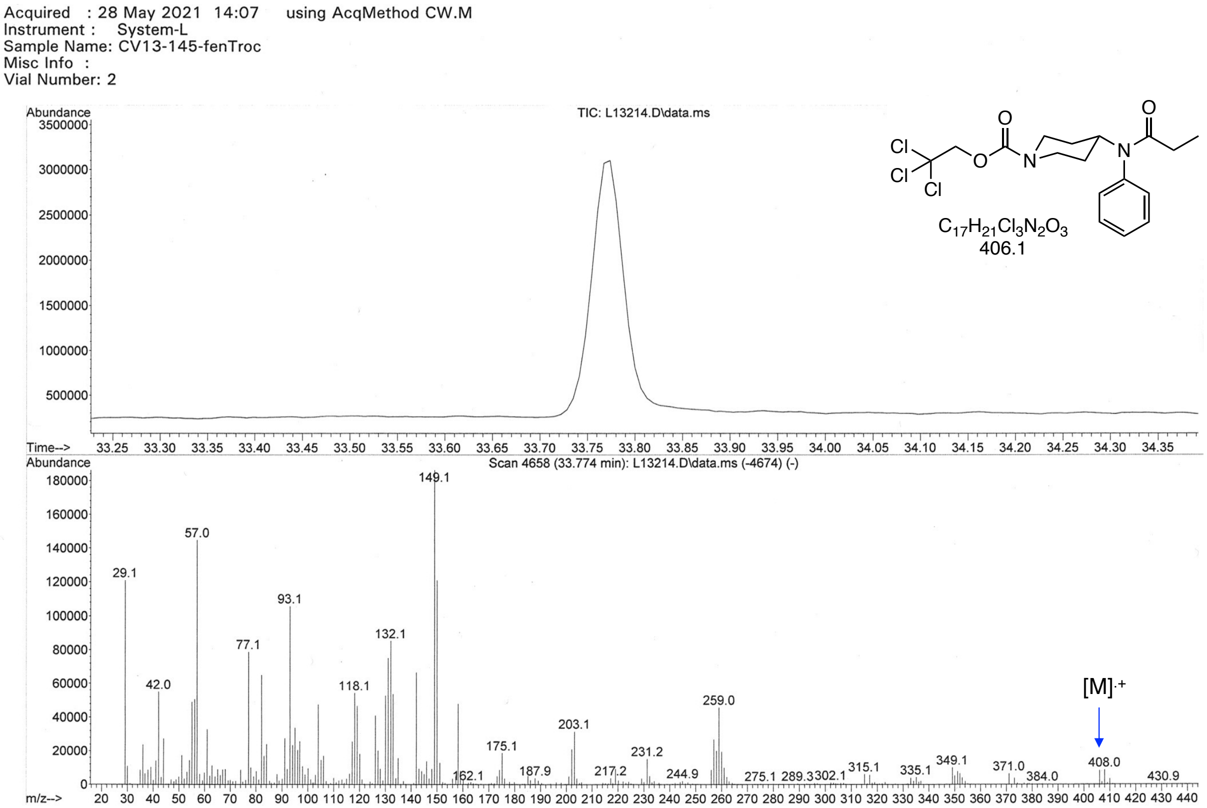

Figure S2. GC-MS chromatogram and MS for Troc-norfentanyl.

Acquired : 31 May 2021 16:02 using AcqMethod CW.M
Instrument : System-L
Sample Name: CV13-145-acetylfen
Misc Info :
Vial Number: 3

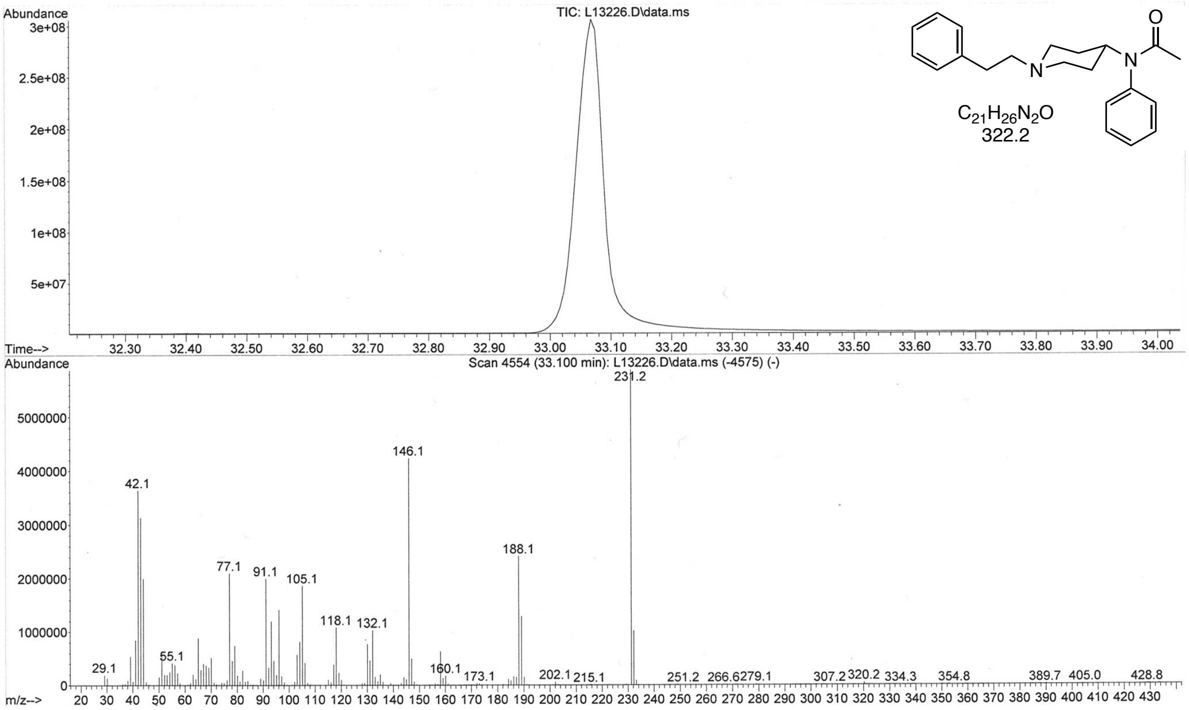

Figure S3. GC-MS chromatogram and MS for acetylfentanyl.

Acquired : 28 May 2021 14:51 using AcqMethod CW.M
Instrument : System-L
Sample Name: CV13-145-acetylfenTroc
Misc Info :
Vial Number: 3

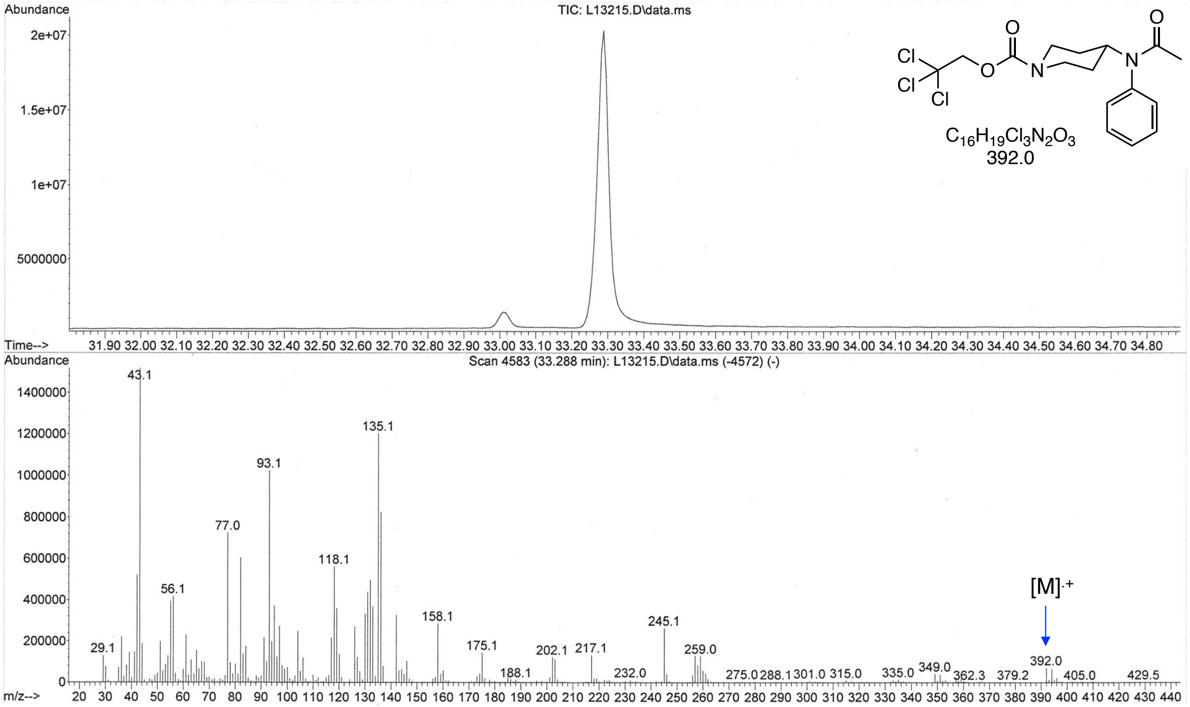

Figure S4. GC-MS chromatogram and MS for Troc-noracetylfentanyl.

Acquired : 31 May 2021 16:47 using AcqMethod CW.M
Instrument : System-L
Sample Name: CV13-145-thiofen
Misc Info :
Vial Number: 4

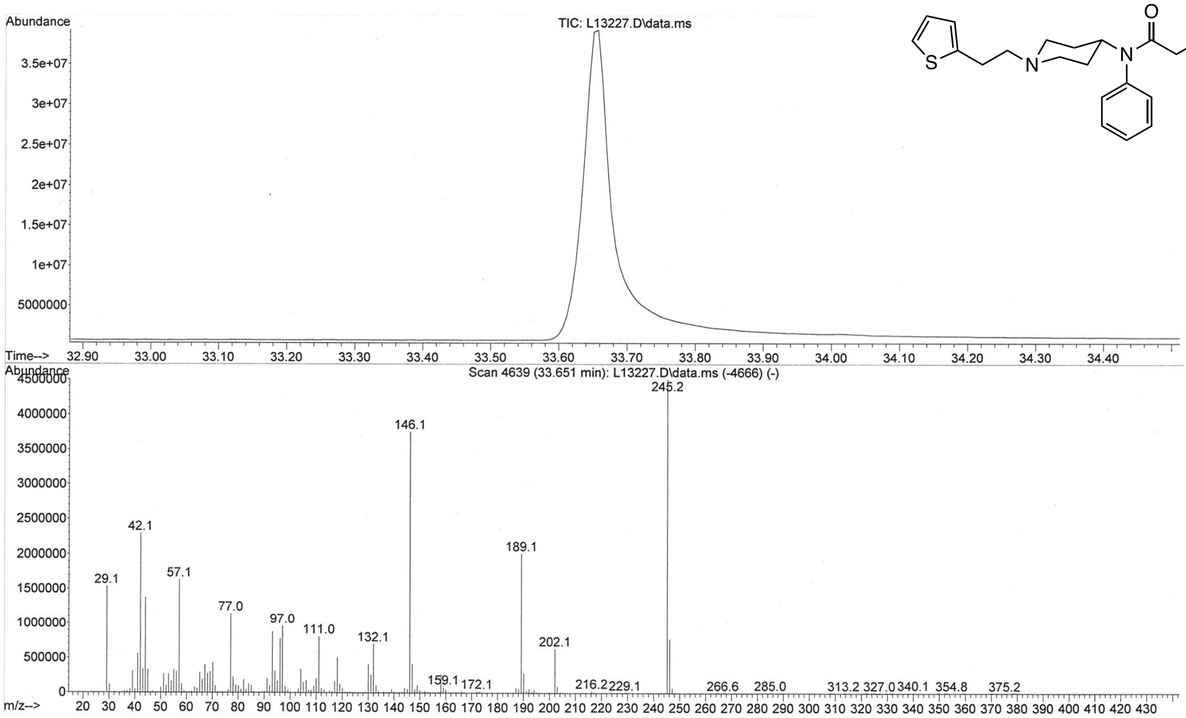

Figure S5. GC-MS chromatogram and MS for thiofentanyl.

Acquired : 28 May 2021 15:35 using AcqMethod CW.M
Instrument : System-L
Sample Name: CV13-145-thiofenTroc
Misc Info :
Vial Number: 4

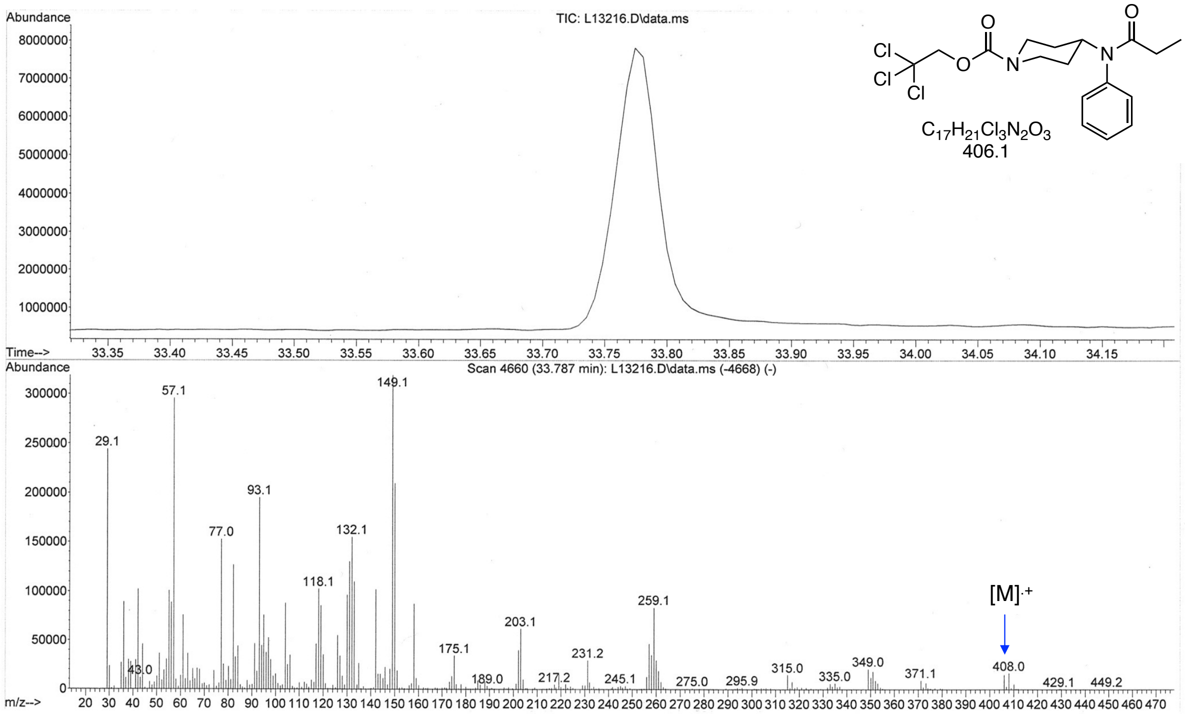

Figure S6. GC-MS chromatogram and MS for Troc-northiofentanyl.

Acquired : 31 May 2021 17:31 using AcqMethod CW.M
Instrument : System-L
Sample Name: CV13-145-butylfen
Misc Info :
Vial Number: 5

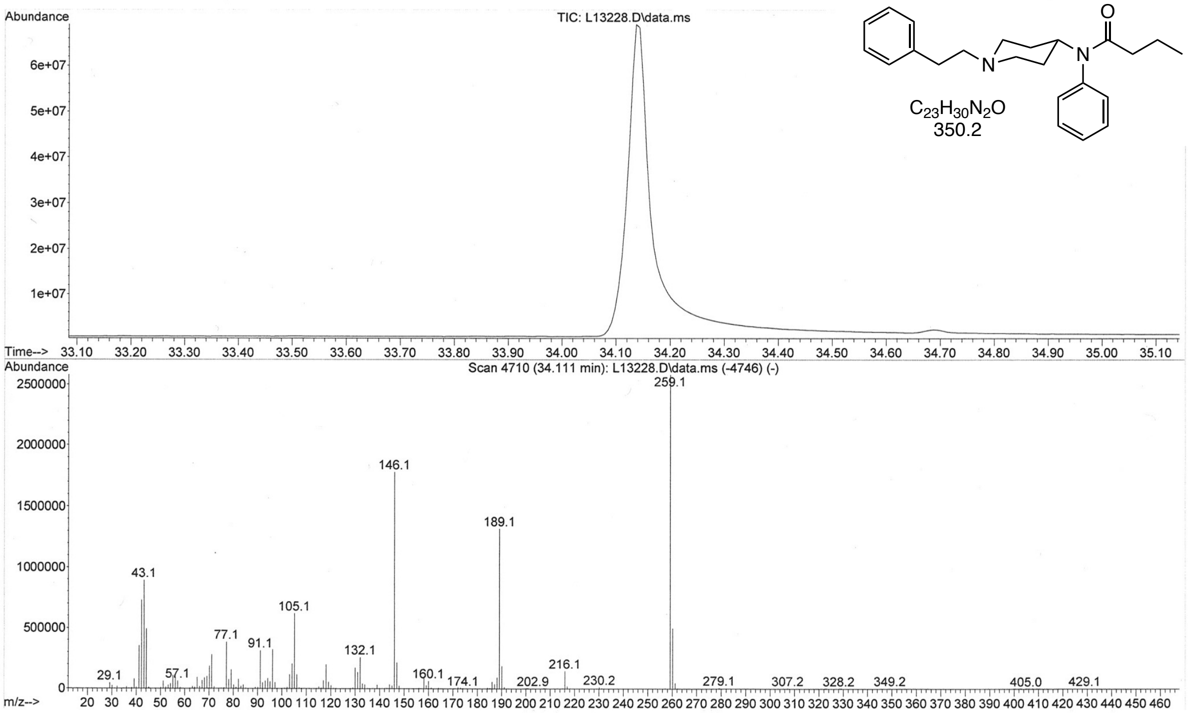

Figure S7. GC-MS chromatogram and MS for butyrylfentanyl.

Acquired : 28 May 2021 16:19 using AcqMethod CW.M
Instrument : System-L
Sample Name: CV13-145-butylfenTroc
Misc Info :
Vial Number: 5

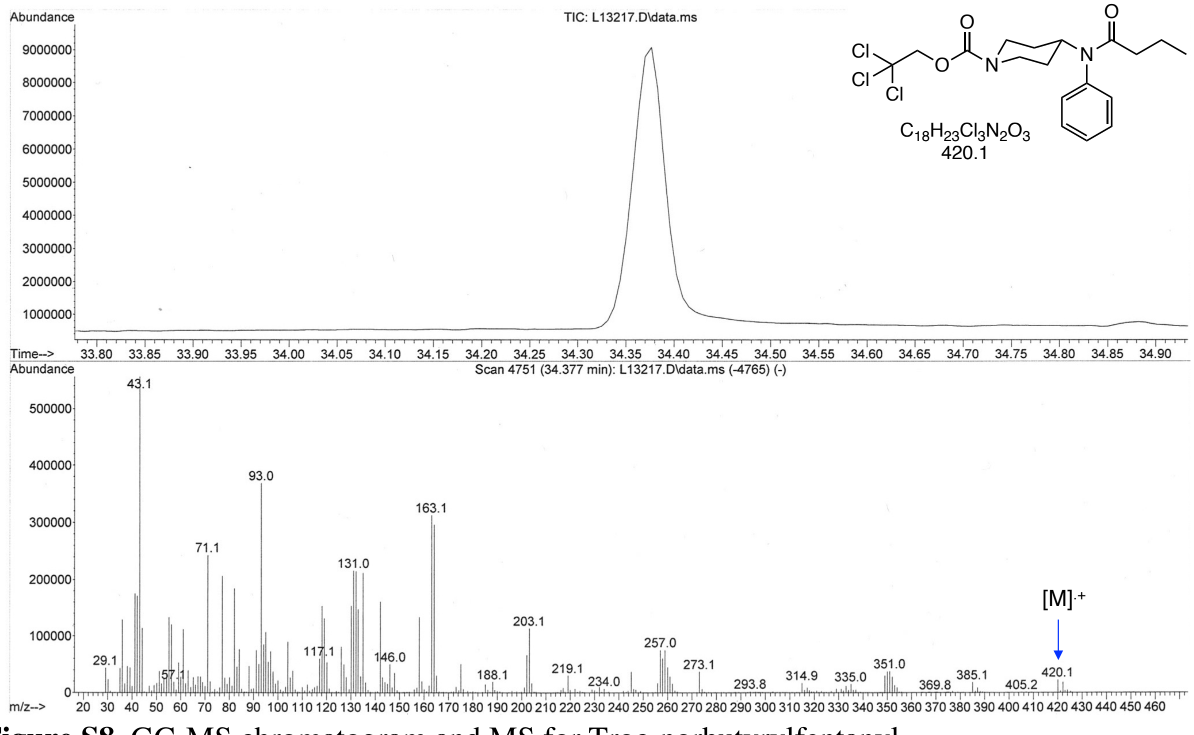

Figure S8. GC-MS chromatogram and MS for Troc-norbutyrylfentanyl.

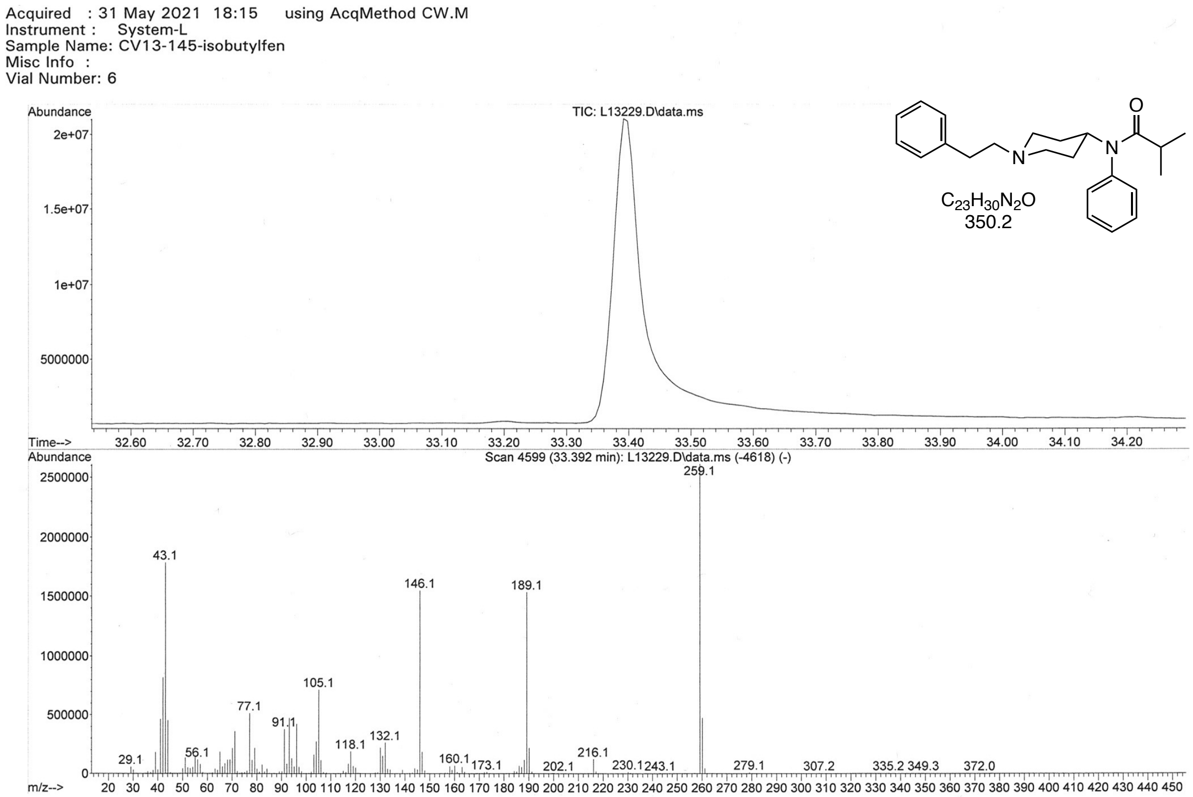

Figure S9. GC-MS chromatogram and MS for isobutyrylfentanyl.

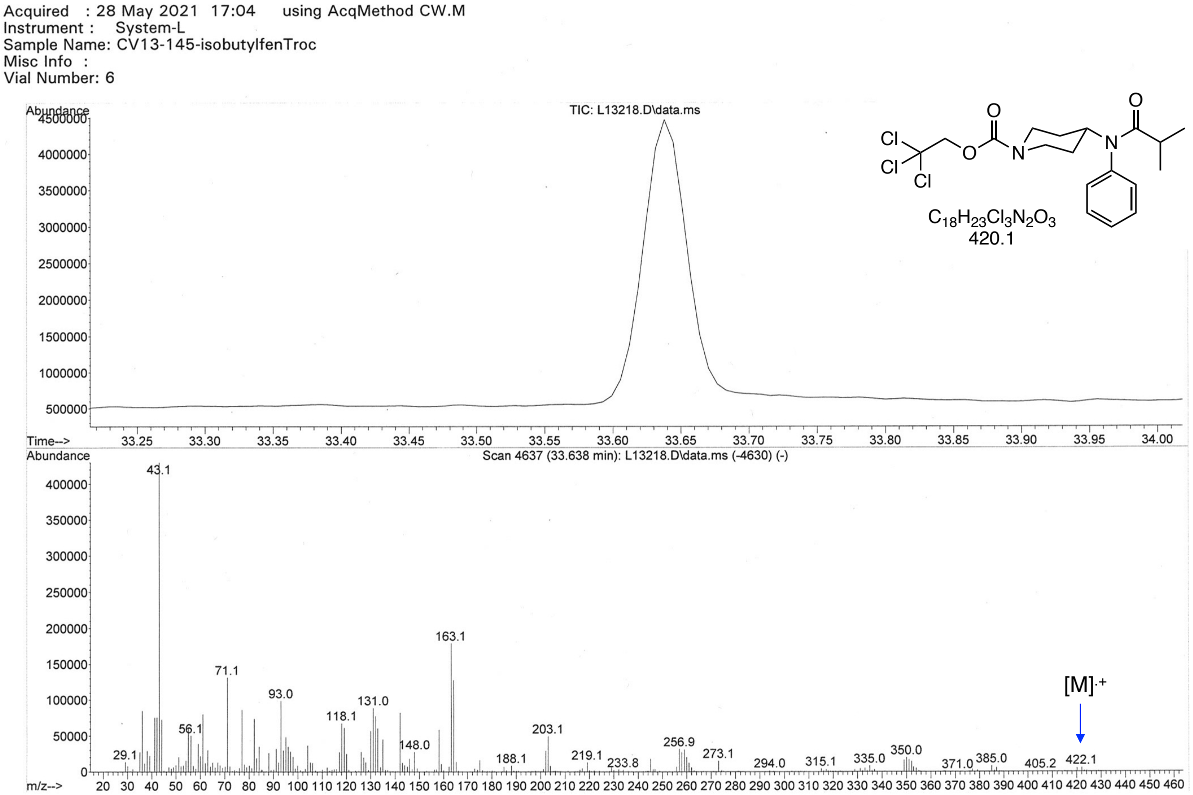

Figure S10. GC-MS chromatogram and MS for Troc-norisobutyrylfentanyl.

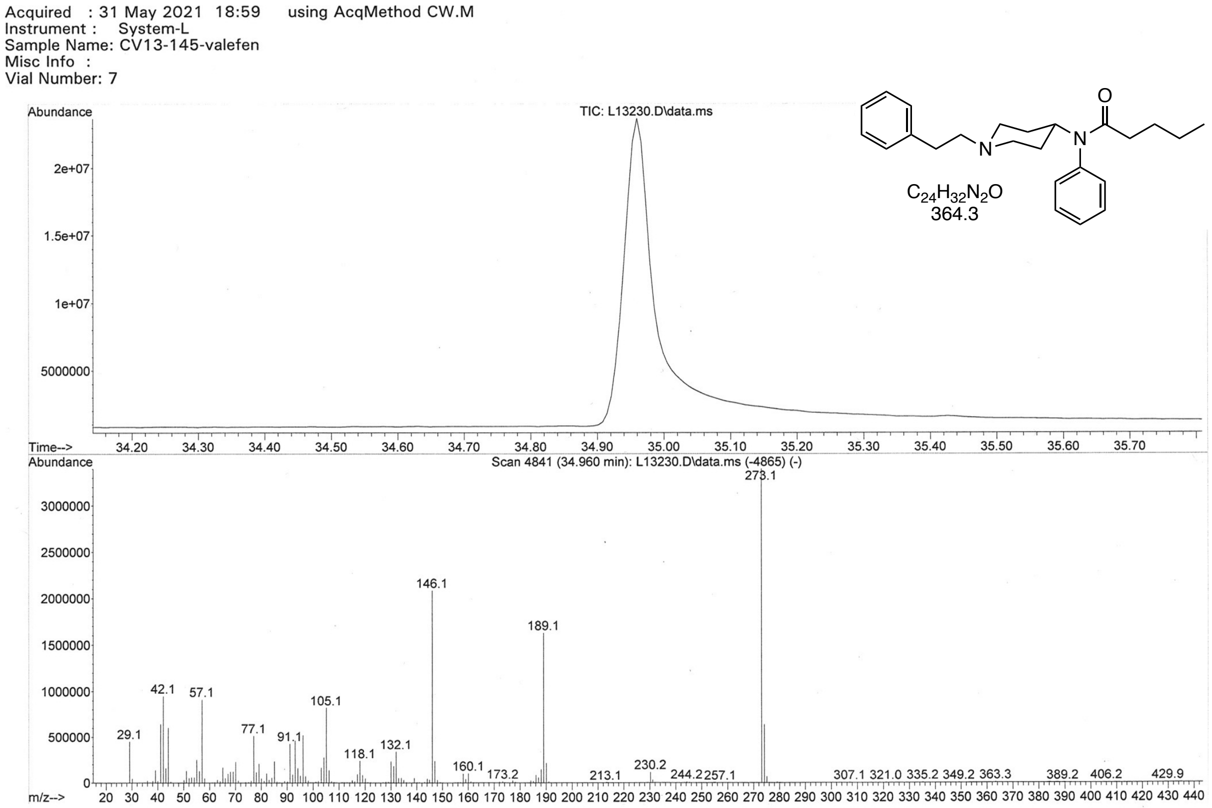

Figure S11. GC-MS chromatogram and MS for valeroylfentanyl.

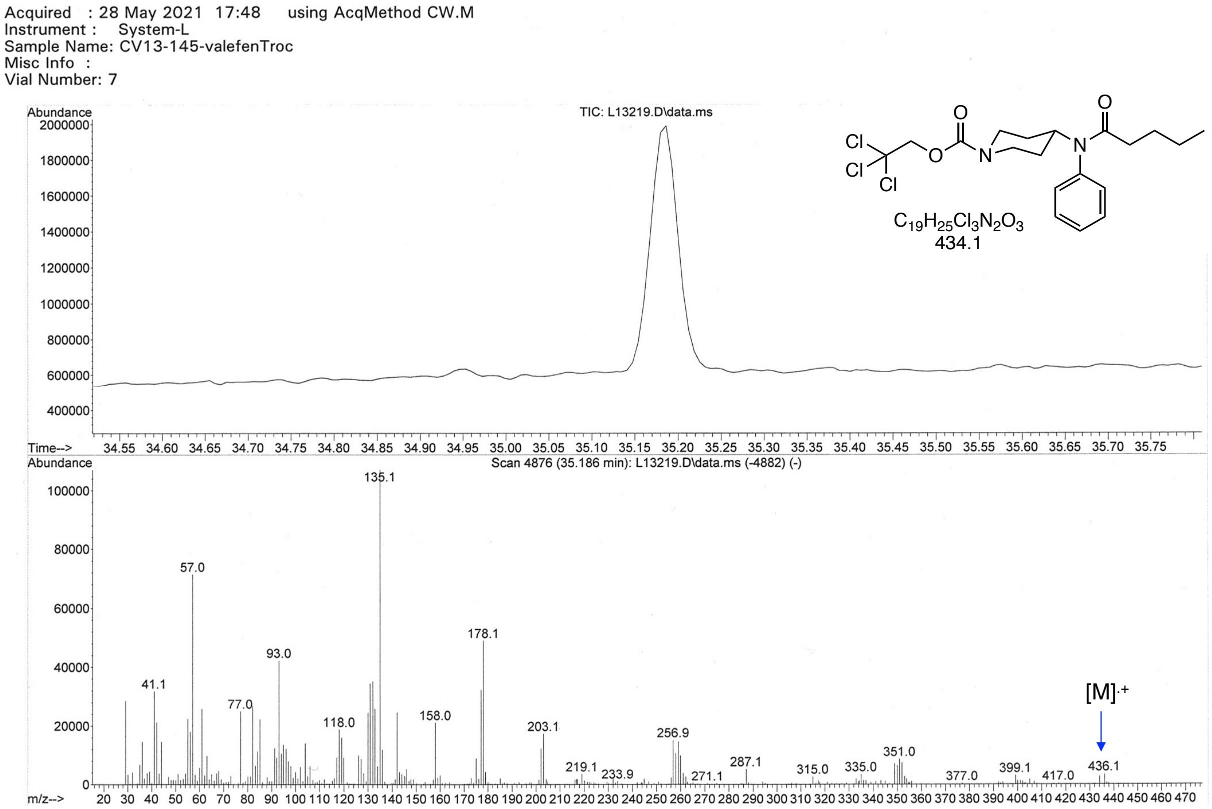

Figure S12. GC-MS chromatogram and MS for Troc-norvaleroylfentanyl.

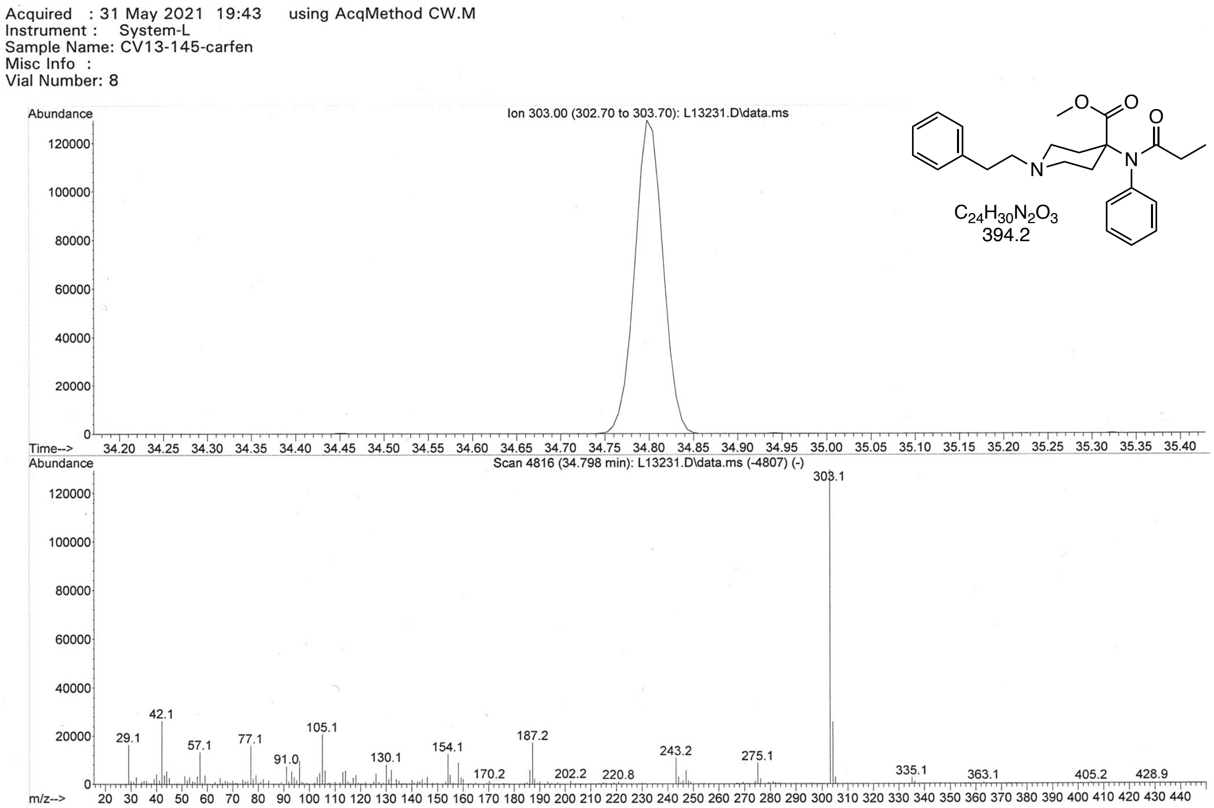

Figure S13. GC-MS chromatogram and MS for carfentanil.

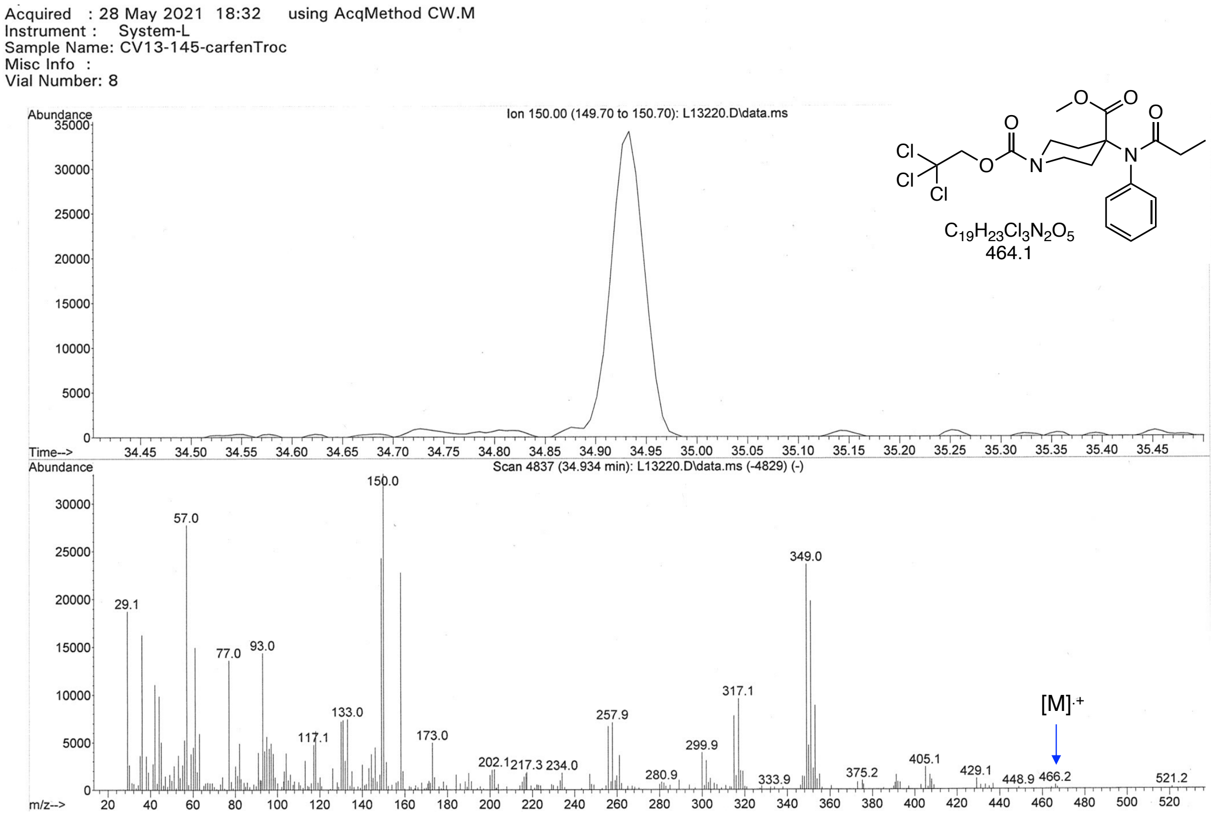

Figure S14. GC-MS chromatogram and MS for Troc-norcarfentanil.

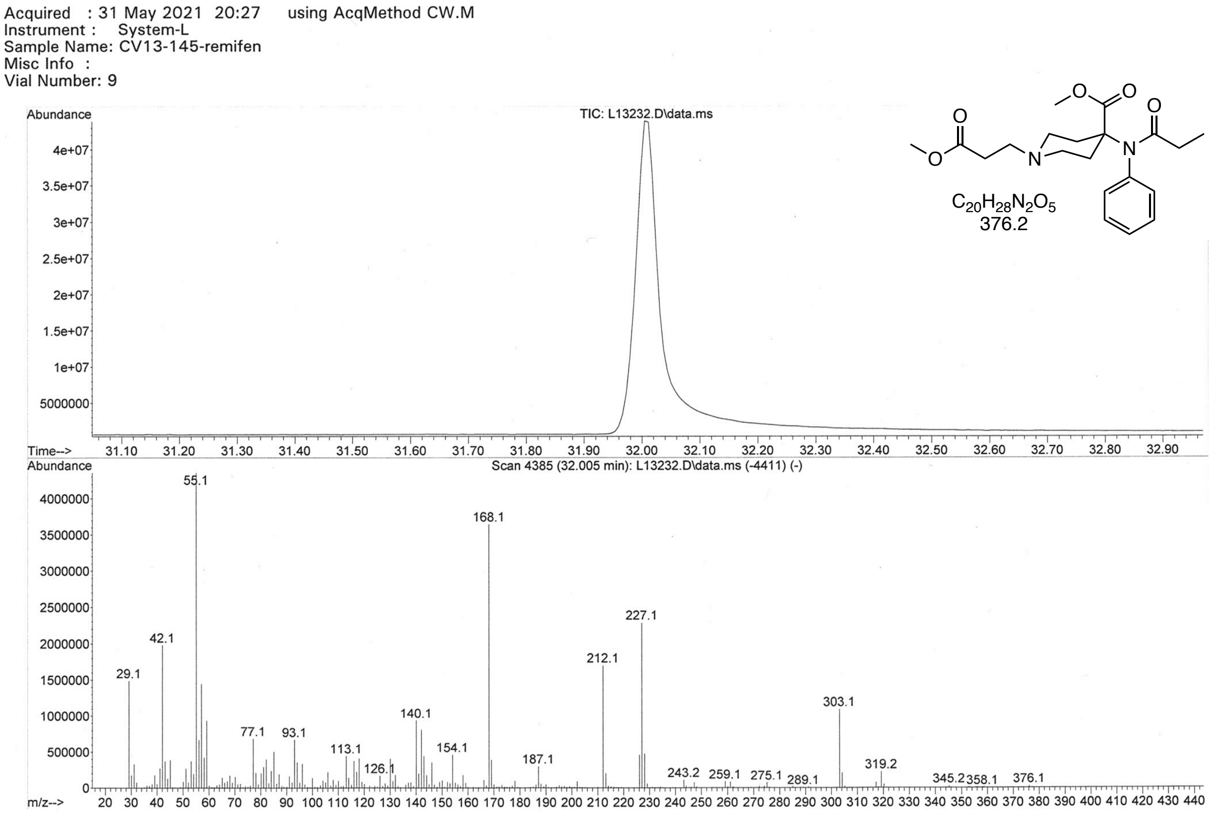

Figure S15. GC-MS chromatogram and MS for remifentanil.

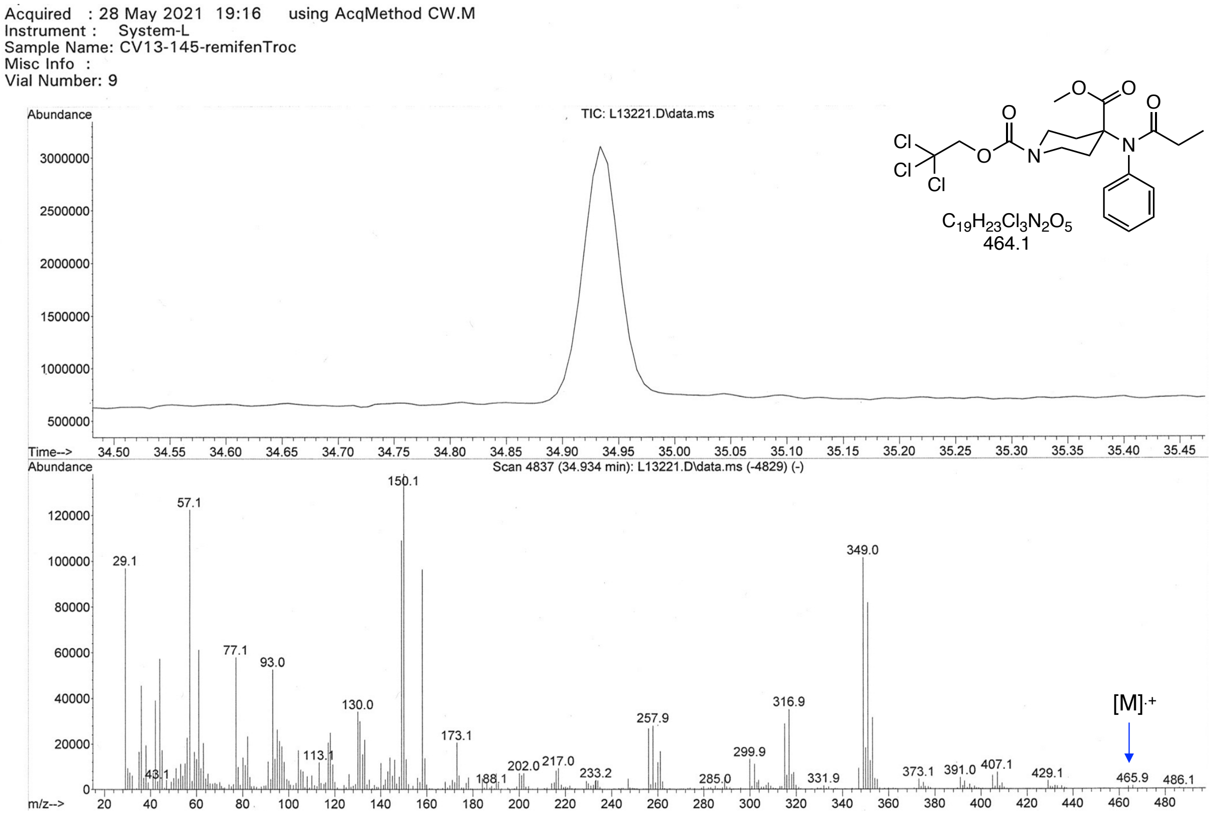

Figure S16. GC-MS chromatogram and MS for Troc-norremifentanil.

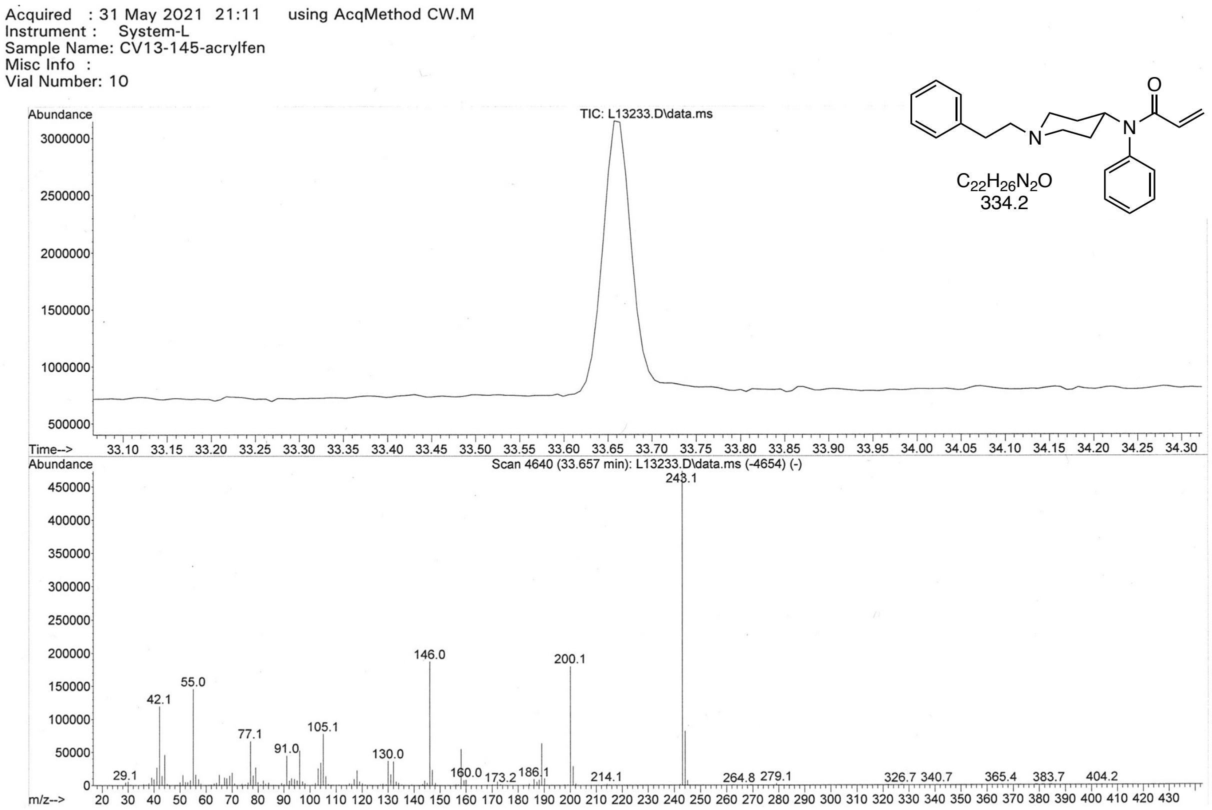

Figure S17. GC-MS chromatogram and MS for acryloylfentanyl.

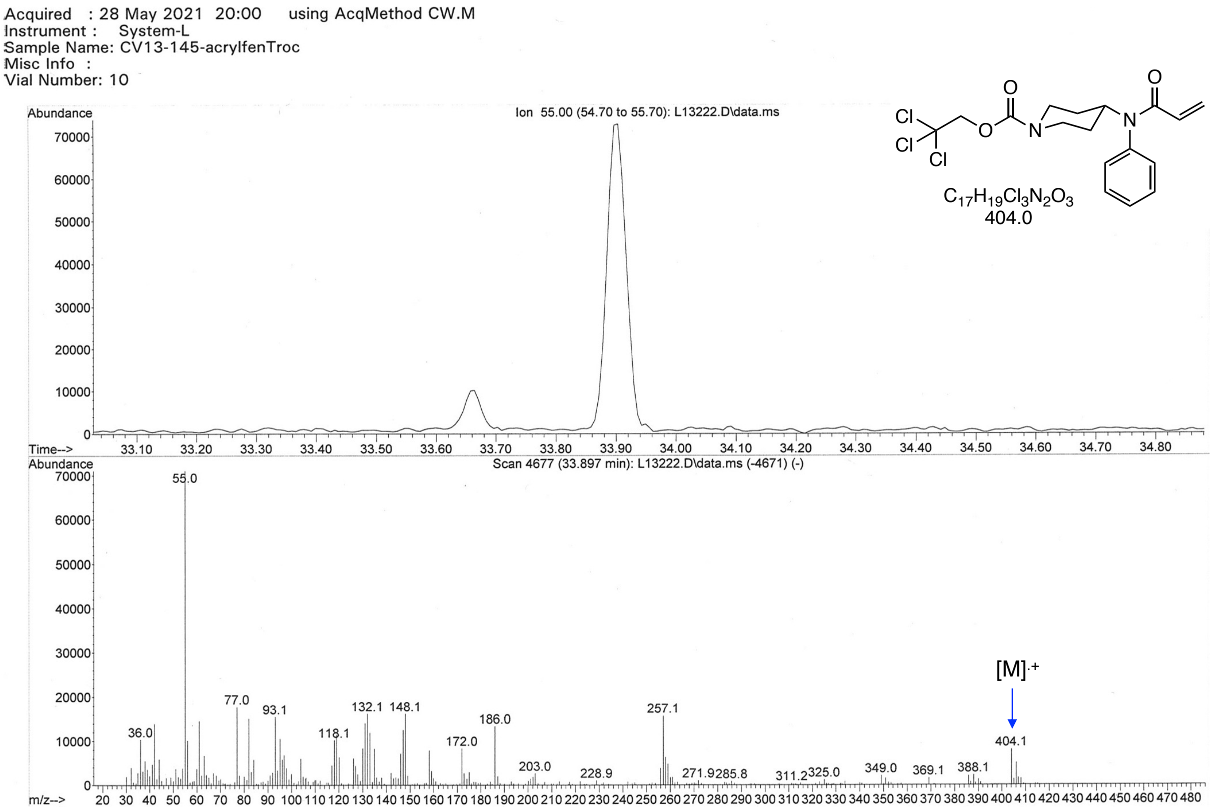

Figure S18. GC-MS chromatogram and MS for Troc-noracryloylfentanyl.

Nuclear Magnetic Resonance. Spectra were obtained using a Bruker Avance III 600 MHz instrument equipped with a Bruker QNP 5 mm cryoprobe (Bruker Biospin, Billerica, MA) at 30.0 ± 0.1°C. NMR data is reported as follows: chemical shift (δ) (parts per million, ppm); multiplicity: m (multiplet), d (doublet), t (triplet), q (quartet), app t (apparent triplet), tt (triplet of triplets), qd (quartet of doublets), sep (septet); coupling constants (*J*) are given in Hertz (Hz). ^{1}H NMR (600 MHz) chemical shifts are calibrated with respect to residual DMSO-$_{d5}$ in DMSO-$_{d6}$ centered at 2.50 ppm, whereas for ^{13}C NMR (151 MHz), the center peak for DMSO-$_{d6}$, centered at 39.52 ppm, was used for the spectral calibration. For acquisitions in $CDCl_3$, chemical shifts are calibrated with respect to residual chloroform in $CDCl_3$ centered at 7.26 ppm, whereas for ^{13}C NMR the center peak for $CDCl_3$, centered at 77.0 ppm, was used for the spectral calibration. ^{13}C-DEPT-135 NMR was used to identify the nature (*i.e.* 1°, 2°, 3° or quaternary) of the carbon atoms in the synthesized targets.

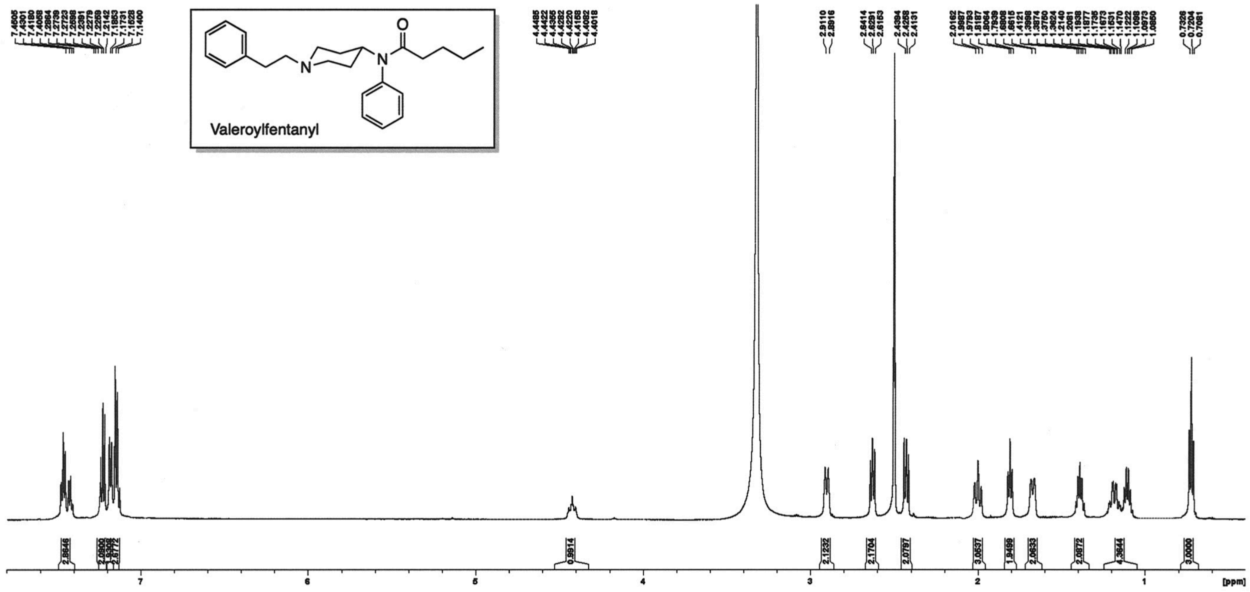

Figure S19. ^{1}H NMR spectrum of valeroylfentanyl (DMSO-$_{d6}$, 600 MHz).

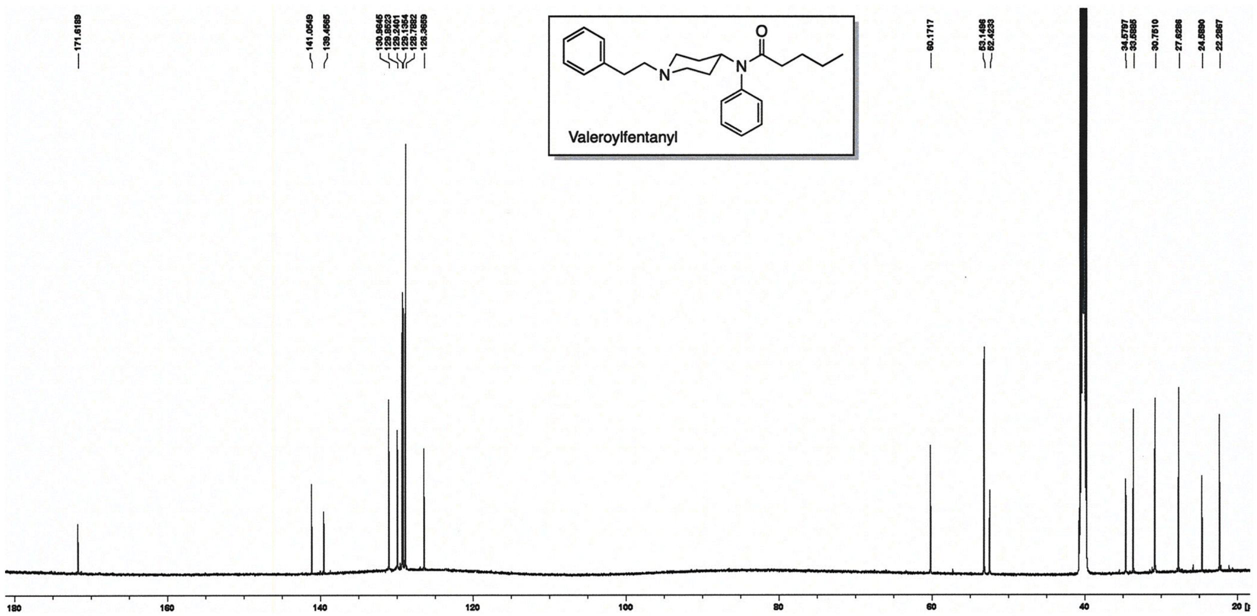

Figure S20. ^{13}C NMR spectrum of valeroylfentanyl (DMSO-$_{d6}$, 151 MHz).

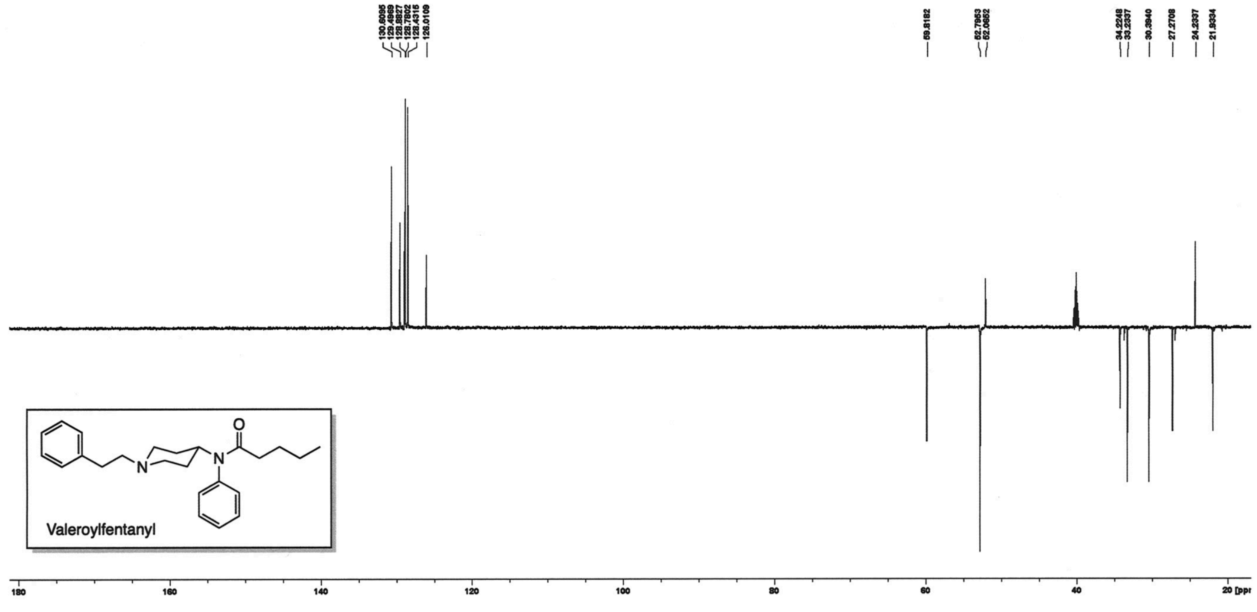

Figure S21. ^{13}C-DEPT-135 NMR spectrum of valeroylfentanyl (DMSO-$_{d6}$, 151 MHz).

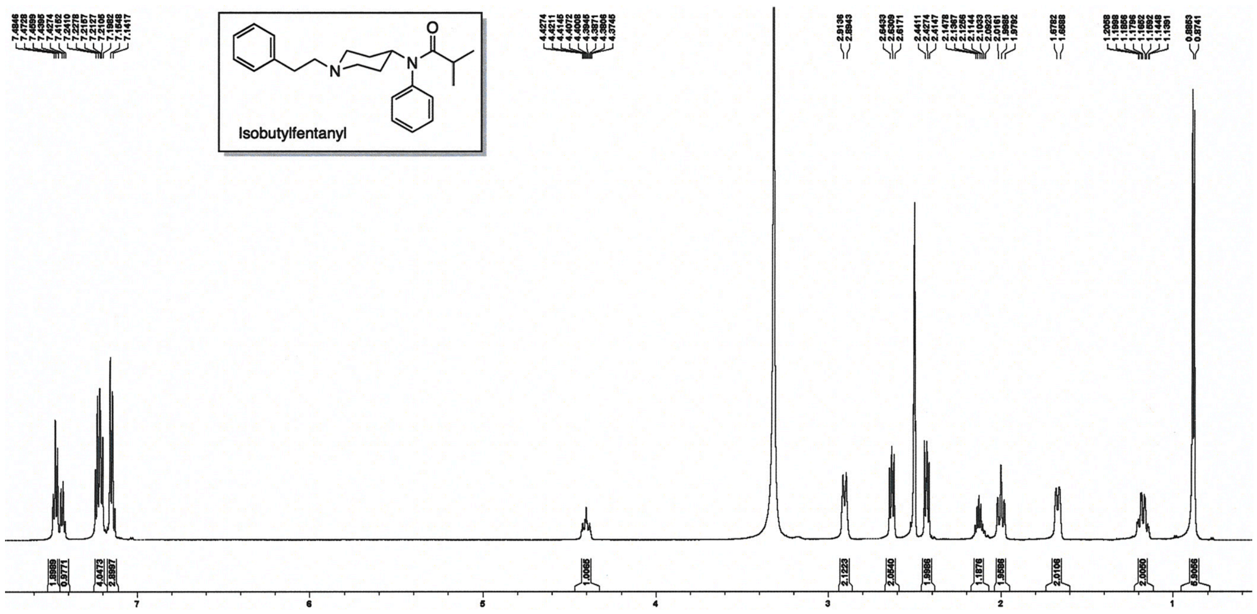

Figure S22. ^{1}H NMR spectrum of isobutyrylfentanyl (DMSO-$_{d6}$, 600 MHz).

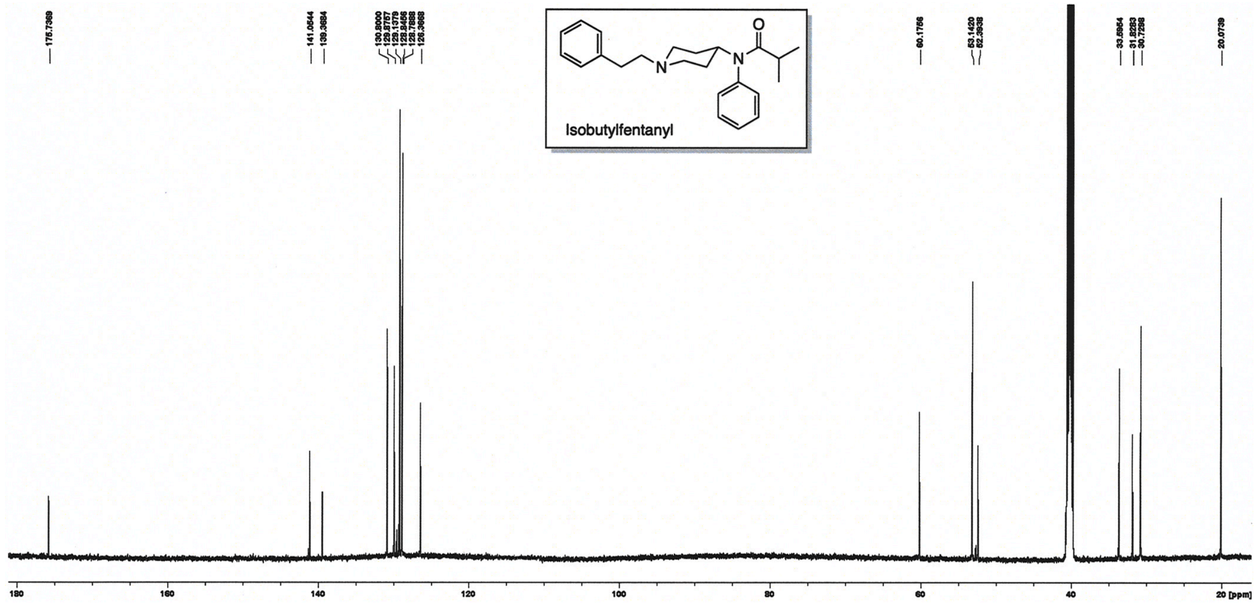

Figure S23. ^{13}C NMR spectrum of isobutyrylfentanyl (DMSO-$_{d6}$, 151 MHz).

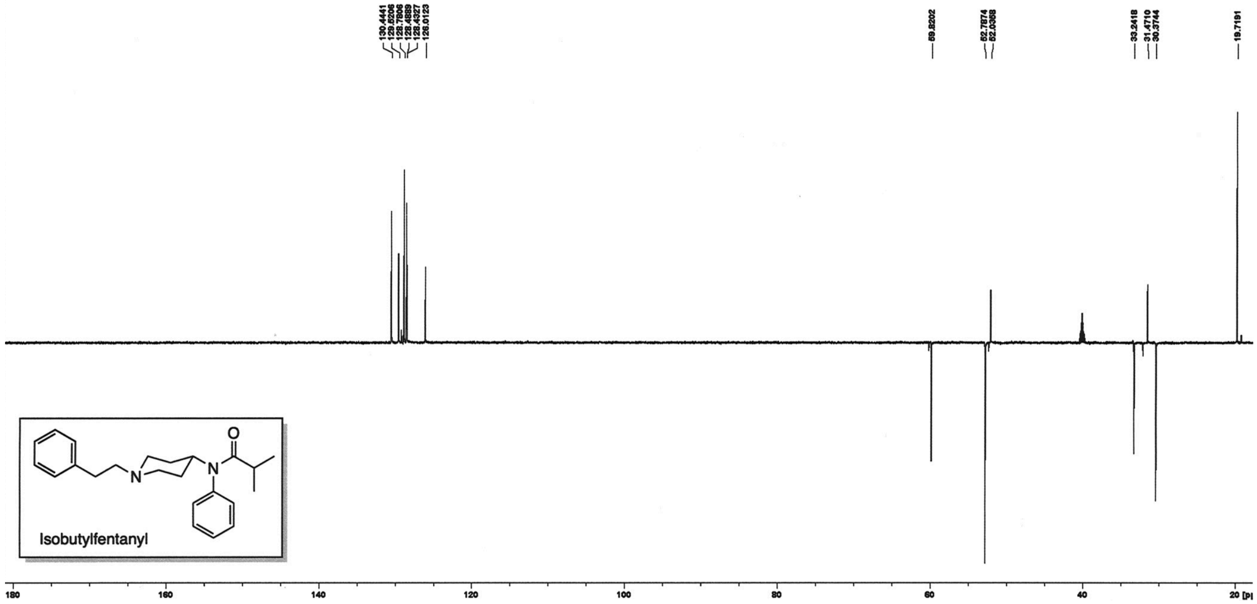

Figure S24. ^{13}C-DEPT-135 NMR spectrum of isobutyrylfentanyl (DMSO-$_{d6}$, 151 MHz).

Synthesis of Troc-norfentanyl

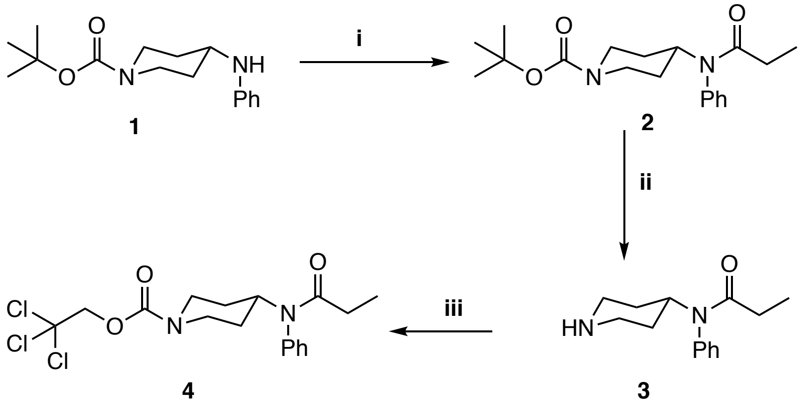

Scheme S1. i) Propanoyl chloride, TEA, DCM, 4 °C → RT (92%); ii) HCl/Et_2O, Et_2O, 4 °C → RT (95%); iii) TrocCl, TEA, 4 °C → RT (81%).

***tert*-butyl 4-(*N*-phenylpropionamido)piperidine-1-carboxylate (2).** *tert*-butyl 4-(phenylamino)piperidine-1-carboxylate **1** (3.0 g, 10.87 mmol) was dissolved in anhydrous DCM (40 mL) in a 100-mL round bottomed flask equipped with a stir bar. The solution was cooled to ~ 4 °C with an ice bath and treated with triethylamine (2.22 mL, 16.3 mmol, 1.5 equiv. to **1**) and stirred for 2 minutes at the end of which time, propanoyl chloride (1.44 mL, 16.3 mmol, 1.5 equiv. to **1**) was added via syringe dropwise over 1 minute. The resulting mixture was stirred at ambient temperature overnight. The following day, the mixture was poured into a 250 mL separatory funnel and partitioned (H_2O//DCM). The organic phase was washed with brine (NaCl/H_2O, 1 x 50 mL), dried over anhydrous sodium sulfate and evaporated *in vacuo* to give a brown oily residue that was purified by flash column chromatography (DCM → 10% MeOH/DCM) to give **2** as an off-white solid (3.32 g, 92%). ^{1}H NMR (600 MHz, $CDCl_3$) δ 7.42-7.38 (m, 3H), 7.06 (d, *J* = 6.4, 2H), 4.79-4.74 (m, 1H), 4.10 (d, *J* = 13.3, 2H), 2.78 (t, *J* = 14.4, 2H), 1.92 (q, *J* = 7.4, 2H), 1.76 (d, *J* = 13.3, 2H), 1.38 (s, 9H), 1.22-1.19 (m, 2H), 1.01 (t, *J* = 7.4, 3H); ^{13}C NMR (151 MHz, $CDCl_3$) δ 173.7, 154.7, 138.9, 130.4, 129.5, 128.6, 79.7, 52.4, 43.4, 30.7, 28.5, 9.73.

***N*-phenyl-*N*-(piperidin-4-yl)propionamide (3).** *tert*-butyl 4-(*N*-phenylpropionamido)piperidine-1-carboxylate **2** (3.32 g, 10.0 mmol) was taken up in diethyl ether (70 mL) in a 250-mL round bottomed flask equipped with a stir bar. The solution was cooled to ~ 4 °C with an ice bath and then using an addition funnel, 2 N HCl in diethyl ether (20 mL, 40.0

mmol, 4 equiv. to **3**) was added dropwise over 5 minutes at ambient temperature. The mixture was stirred vigorously overnight. The following day, 1 N NaOH (50 mL) was added to the white suspension and the mixture was transferred to a 250 mL separatory funnel. The organic phase was extracted with 1 N NaOH/H_2O (2 x 50 mL), dried over anhydrous sodium sulfate and evaporated *in vacuo* to give **3** as a light-yellow solid of high purity (2.21 g, 95%). The solid was taken directly onto the next step. ^{1}H NMR (600 MHz, $CDCl_3$) δ 7.38-7.33 (m, 3H), 7.05-7.03 (m, 2H), 4.73-4.68 (m, 1H), 3.05-3.02 (m, 2H), 2.69 (t, *J* = 12.4, 2H), 2.53 (br s, 1H), 1.89 (q, *J* = 7.4, 2H), 1.77-1.75 (m, 2H), 1.28-1.22 (m, 2H), 0.97 (t, *J* = 7.4, 3H); ^{13}C NMR (151 MHz, $CDCl_3$) δ 173.5, 139.0, 130.5, 129.4, 128.4, 52.3, 46.1, 31.7, 28.6, 9.72.

Troc-norfentanyl (**4**). *N*-phenyl-*N*-(piperidin-4-yl)propionamide **3** (2.1 g, 9.1 mmol) was dissolved in anhydrous DCM (50 mL) in a 250-mL round bottomed flask equipped with a stir bar. The solution was cooled to ~ 4 °C with an ice bath and to the pale-yellow solution, triethylamine (1.58 mL, 11.3 mmol, 1.25 equiv. to **3**) was added via syringe followed by the addition of TrocCl (1.55 mL, 11.3 mmol, 1.25 equiv. to **3**) via syringe. The resulting mixture was vigorously stirred at ambient temperature overnight. The following day, the mixture was transferred to a 250 mL separatory funnel and partitioned (H_2O//DCM). The organic phase was washed with brine (NaCl/H_2O, 2.x 50 mL), dried over anhydrous sodium sulfate and evaporated *in vacuo* to give a yellow residue that was purified by flash column chromatography (DCM → 20% MeOH/DCM) to give **4** as an off-white solid (3.0 g, 81%). ^{1}H NMR (600 MHz, $CDCl_3$) δ 7.43-7.37 (m, 3H), 7.06-7.04 (m, 2H), 4.88-4.82 (m, 1H), 4.68-4.65 (m, 2H), 4.22 (d, *J* = 13.5, 2H), 3.01-2.88 (m, 2H), 1.93 (q, *J* = 7.4, 2H), 1.84-1.82 (m, 2H), 1.32-1.26 (m, 2H), 1.01 (t, *J* = 7.4, 3H) ; ^{13}C NMR (151 MHz, $CDCl_3$) δ 173.7, 153.4, 138.7, 130.4, 129.6, 128.7, 95.8, 75.1, 52.0, 44.2, 43.9, 30.7, 30.4, 28.6, 9.69.

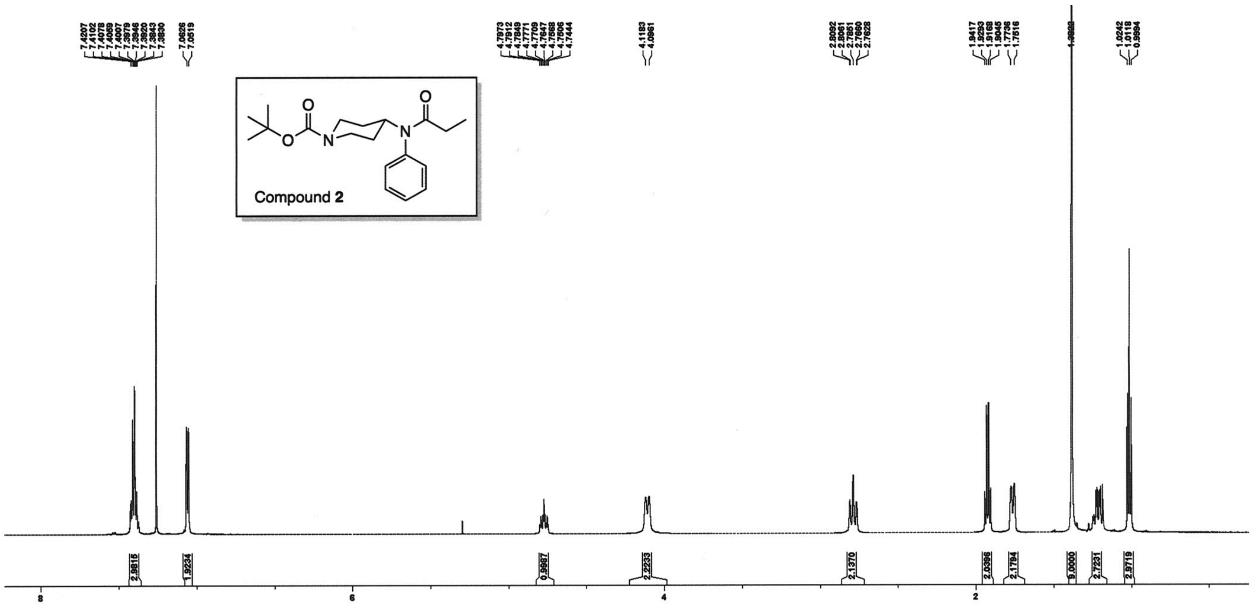

Figure S25. ^{1}H NMR spectrum of compound **2**.

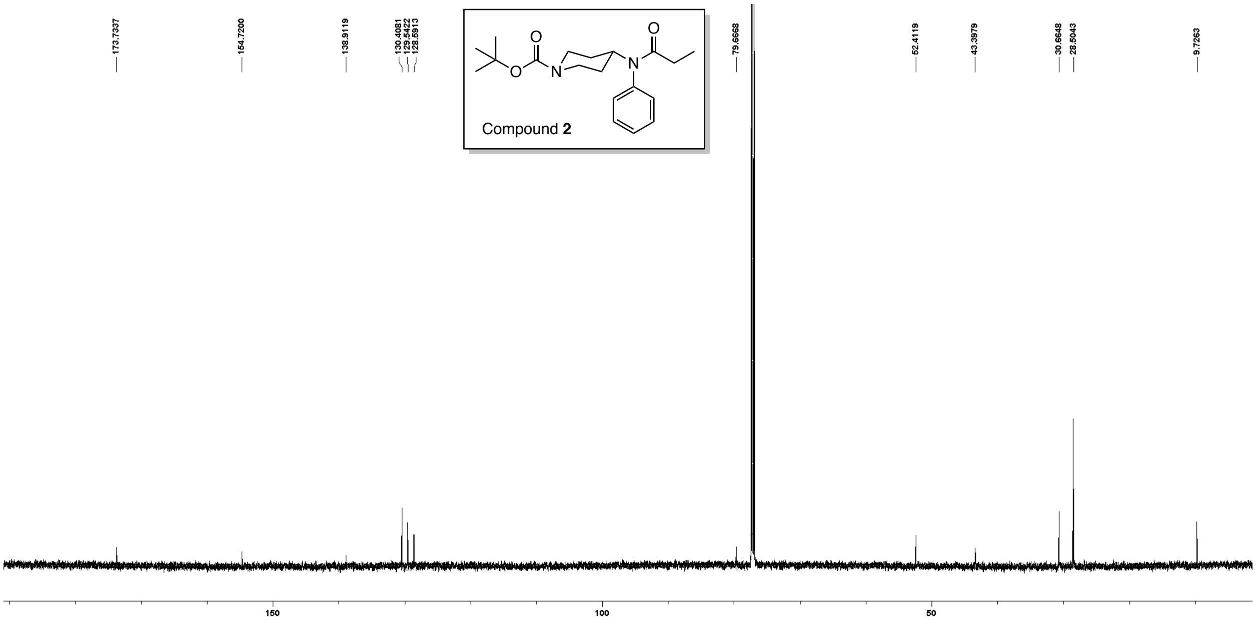

Figure S26. ^{13}C NMR spectrum of compound **2**.

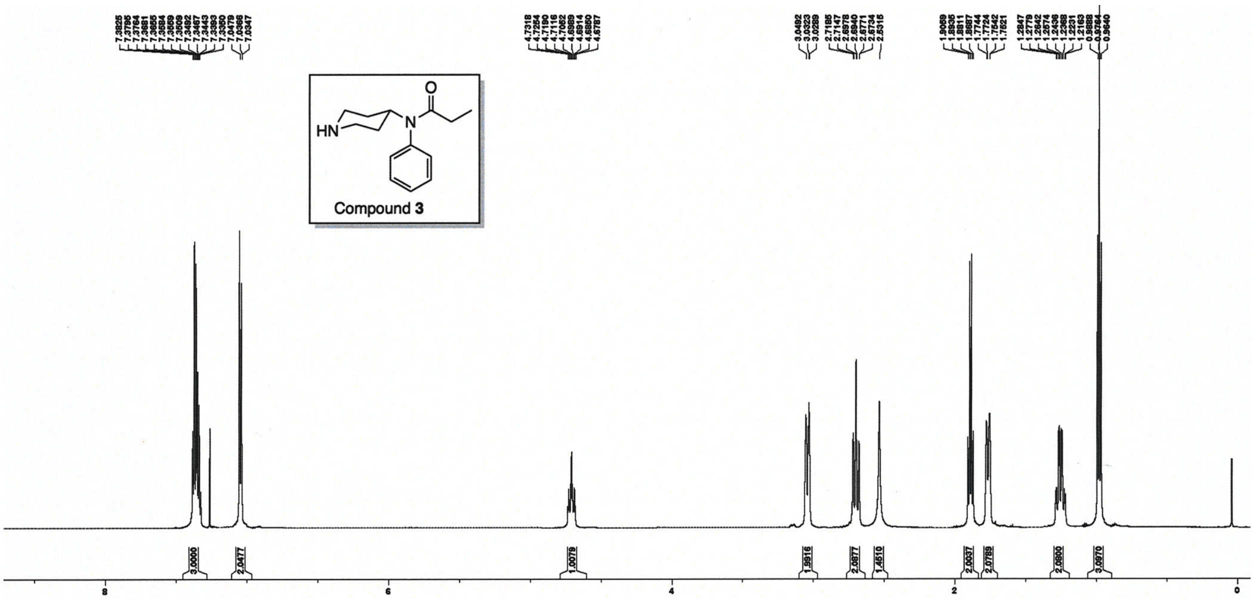

Figure S27. ^{1}H NMR spectrum of compound **3**.

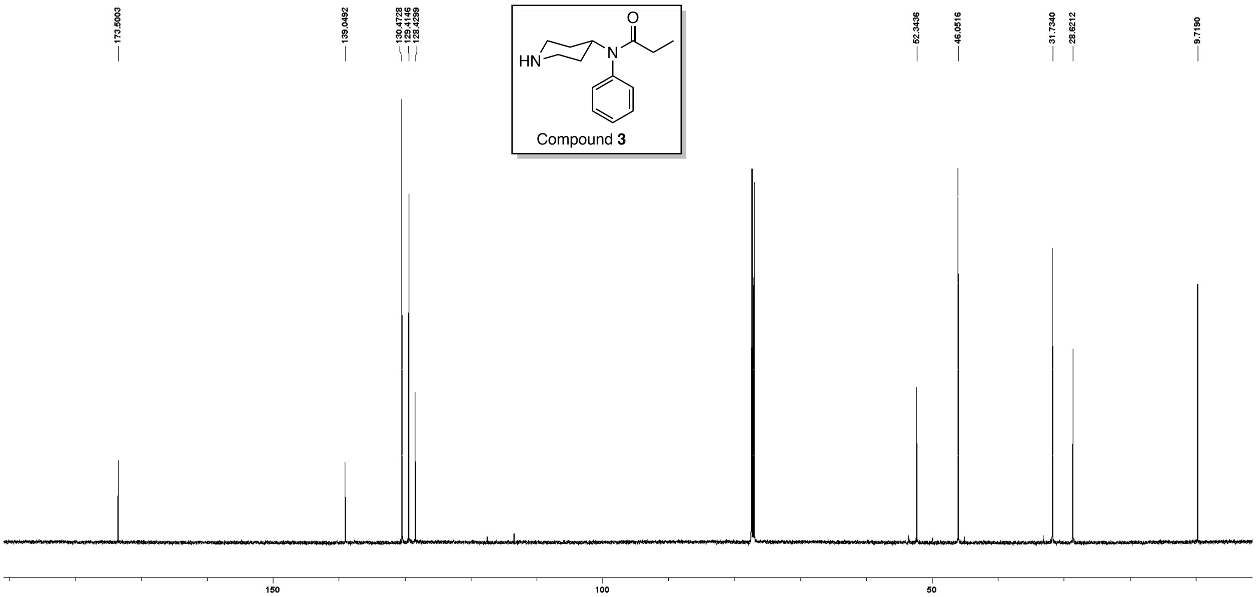

Figure S28. ^{13}C NMR spectrum of compound **3**.

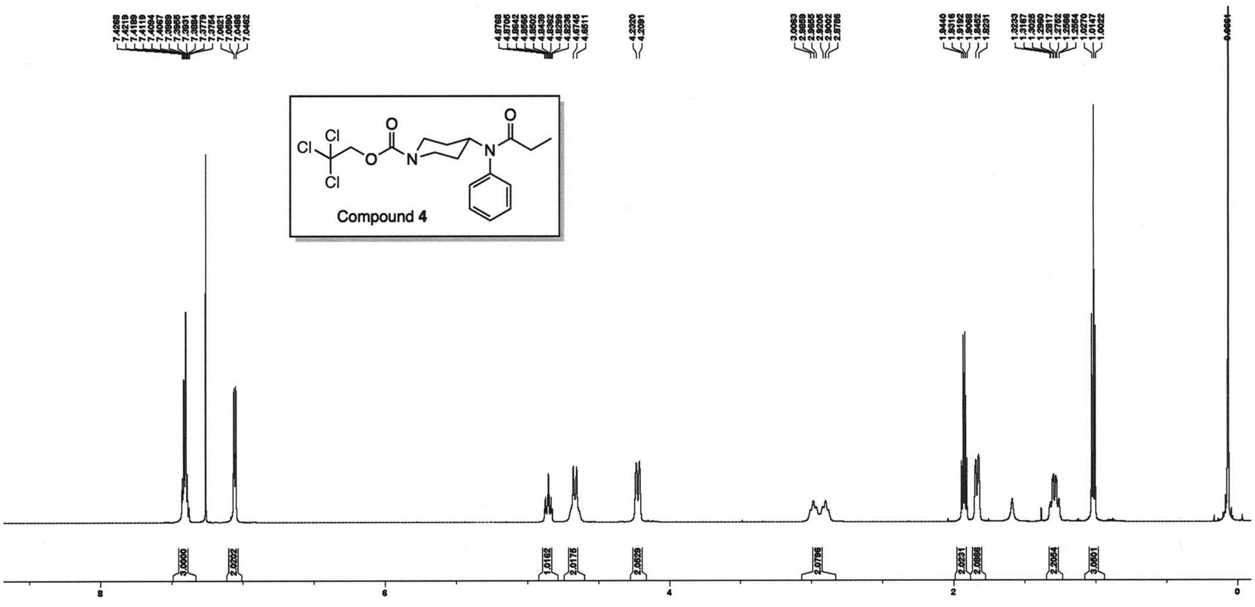

Figure S29. ^{1}H NMR spectrum of Troc-norfentanyl.

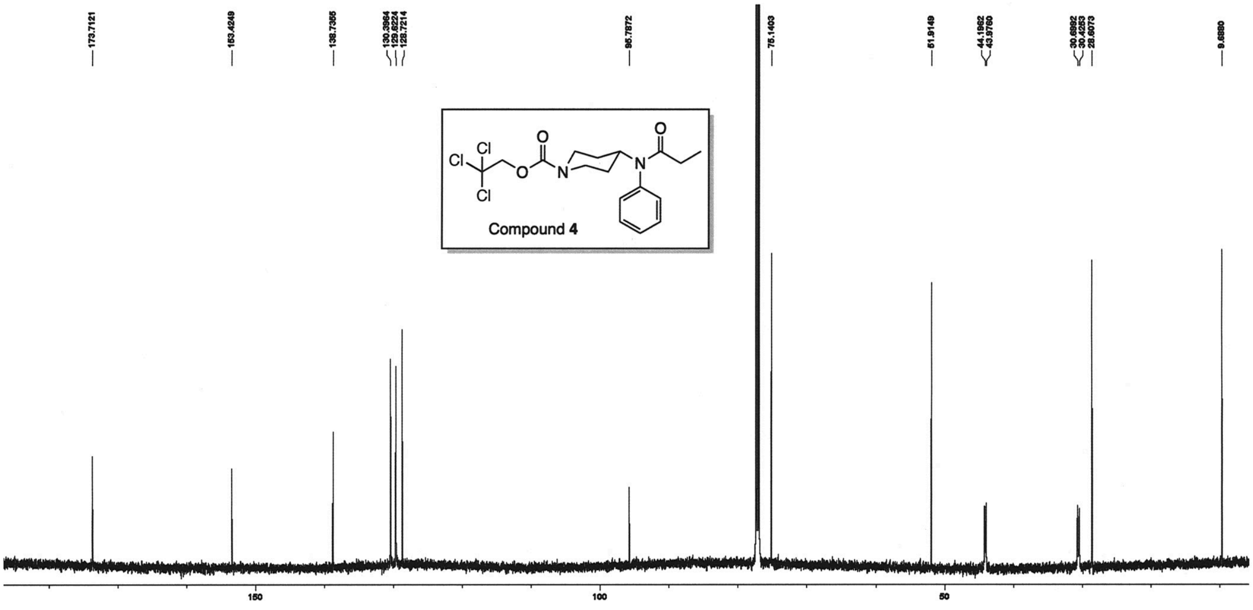

Figure S30. ^{13}C NMR spectrum of Troc-norfentanyl.

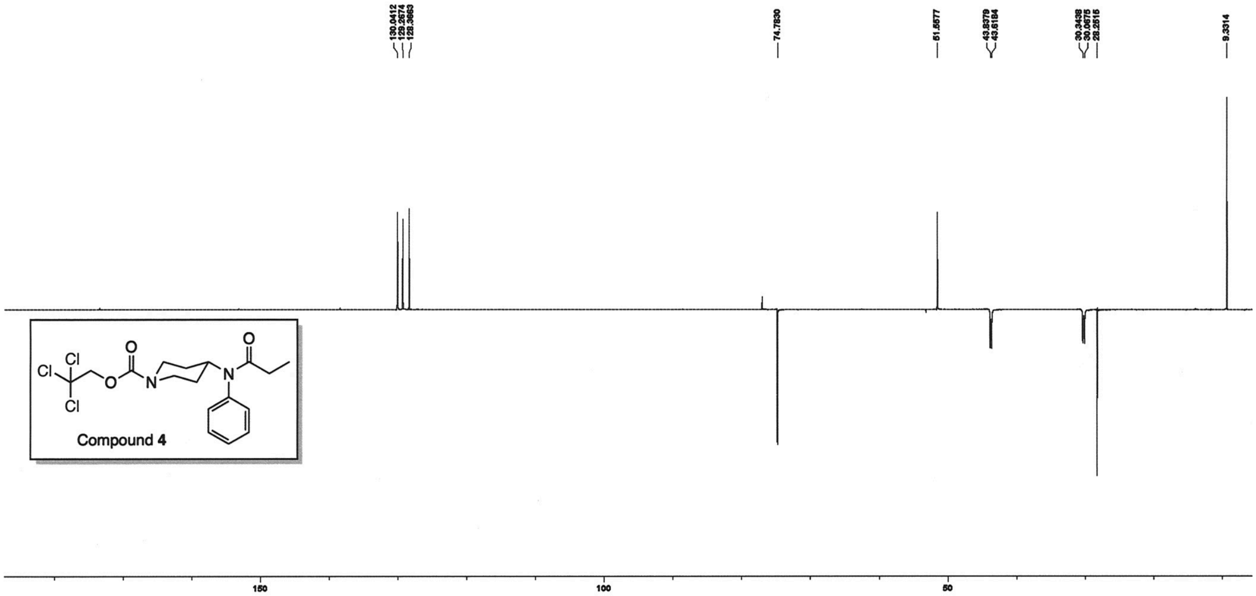

Figure S31. ^{13}C-DEPT-135 NMR spectrum of Troc-norfentanyl.

GCMS data (raw) used for calculating the LLOD and LLOQ for the method (using Troc-norfentanyl)

Signal to Noise Report

Data Path : C:\gcms\1\data\LOD and LOQ Troc-Norfent System-Y\
Data File : Y3381.D
Acq On : 21 Sep 2021 14:26
Operator :
Sample : LOG-FSC-203-48-2
Misc : 0.020 ug/mL Troc-Norfentanyl
ALS Vial : 1 Sample Multiplier: 1

Integration File: events.e

Method : C:\gcms\1\methods\default.m
Title :
Last Update :

Signal Used: TIC; data.ms

Signal region: 8.32 to 8.33 min; height: 15699
Noise region : 8.27 to 8.30 min; Max noise 10900.0, Min noise 10536.0

Calculations	Value
Noise Points used	8
Average noise = (sum of noise)/points	10701.4
Corrected Signal = height/Average noise	4997.6
Pk-pk noise = Max noise/Min noise	364.0
Pk-pk S/N = Corrected signal/Pk-pk noise	13.7
RMS noise = SQRT(sum(square(noise-avg noise))/points)	125.3
RMS S/N = Corrected signal/RMS noise	39.9

default.m Fri Sep 24 15:01:30 2021 SYSTEM-Y

Signal to Noise Report

Data Path : C:\gcms\1\data\LOD and LOQ Troc-Norfent System-Y\
Data File : Y3382.D
Acq On : 21 Sep 2021 14:39
Operator :
Sample : LOG-FSC-203-48-3
Misc : 0.010 ug/mL Troc-Norfentanyl
ALS Vial : 2 Sample Multiplier: 1

Integration File: events.e

Method : C:\gcms\1\methods\default.m
Title :
Last Update :

Signal Used: TIC; data.ms

Signal region: 8.31 to 8.32 min; height: 12606
Noise region : 8.25 to 8.28 min; Max noise 10484.0, Min noise 9978.0

Calculations	Value
Noise Points used	8
Average noise = (sum of noise)/points	10224.5
Corrected Signal = height/Average noise	2381.5
Pk-pk noise = Max noise/Min noise	506.0
Pk-pk S/N = Corrected signal/Pk-pk noise	4.7
RMS noise = SQRT(sum(square(noise-avg noise))/points)	147.7
RMS S/N = Corrected signal/RMS noise	16.1

default.m Fri Sep 24 15:03:53 2021 SYSTEM-Y

Signal to Noise Report

Data Path : C:\gcms\1\data\LOD and LOQ Troc-Norfent System-Y\
Data File : Y3383.D
Acq On : 21 Sep 2021 14:51
Operator :
Sample : LOG-FSC-203-48-2
Misc : 0.020 ug/mL Troc-Norfentanyl
ALS Vial : 1 Sample Multiplier: 1

Integration File: events.e

Method : C:\gcms\1\methods\default.m
Title :
Last Update :

Signal Used: TIC; data.ms

Signal region: 8.30 to 8.32 min; height: 14732
Noise region : 8.24 to 8.27 min; Max noise 10585.0, Min noise 10313.0

Calculations	Value
Noise Points used	9
Average noise = (sum of noise)/points	10453.3
Corrected Signal = height/Average noise	4278.7
Pk-pk noise = Max noise/Min noise	272.0
Pk-pk S/N = Corrected signal/Pk-pk noise	15.7
RMS noise = SQRT(sum(square(noise-avg noise))/points)	113.8
RMS S/N = Corrected signal/RMS noise	37.6

default.m Fri Sep 24 15:05:20 2021 SYSTEM-Y

Signal to Noise Report

Data Path : C:\gcms\1\data\LOD and LOQ Troc-Norfent System-Y\
Data File : Y3384.D
Acq On : 21 Sep 2021 15:03
Operator :
Sample : LOG-FSC-203-48-3
Misc : 0.010 ug/mL Troc-Norfentanyl
ALS Vial : 2 Sample Multiplier: 1

Integration File: events.e

Method : C:\gcms\1\methods\default.m
Title :
Last Update :

Signal Used: TIC; data.ms

Signal region: 8.30 to 8.32 min; height: 12240
Noise region : 8.25 to 8.28 min; Max noise 10226.0, Min noise 9788.0

Calculations	Value
Noise Points used	8
Average noise = (sum of noise)/points	9930.9
Corrected Signal = height/Average noise	2309.1
Pk-pk noise = Max noise/Min noise	438.0
Pk-pk S/N = Corrected signal/Pk-pk noise	5.3
RMS noise = SQRT(sum(square(noise-avg noise))/points)	132.7
RMS S/N = Corrected signal/RMS noise	17.4

default.m Fri Sep 24 15:07:13 2021 SYSTEM-Y

Signal to Noise Report

Data Path : C:\gcms\1\data\LOD and LOQ Troc-Norfent System-Y\
Data File : Y3385.D
Acq On : 21 Sep 2021 15:15
Operator :
Sample : LOG-FSC-203-48-2
Misc : 0.020 ug/mL Troc-Norfentanyl
ALS Vial : 1 Sample Multiplier: 1

Integration File: events.e

Method : C:\gcms\1\methods\default.m
Title :
Last Update :

Signal Used: TIC; data.ms

Signal region: 8.30 to 8.32 min; height: 14287
Noise region : 8.22 to 8.25 min; Max noise 10033.0, Min noise 9622.0

Calculations	Value
Noise Points used	7
Average noise = (sum of noise)/points	9846.0
Corrected Signal = height/Average noise	4441.0
Pk-pk noise = Max noise/Min noise	411.0
Pk-pk S/N = Corrected signal/Pk-pk noise	10.8
RMS noise = SQRT(sum(square(noise-avg noise))/points)	114.6
RMS S/N = Corrected signal/RMS noise	38.7

default.m Fri Sep 24 15:08:40 2021 SYSTEM-Y

Signal to Noise Report

Data Path : C:\gcms\1\data\LOD and LOQ Troc-Norfent System-Y\
Data File : Y3386.D

Acq On : 21 Sep 2021 15:27
Operator :
Sample : LOG-FSC-203-48-3
Misc : 0.010 ug/mL Troc-Norfentanyl
ALS Vial : 2 Sample Multiplier: 1

Integration File: events.e

Method : C:\gcms\1\methods\default.m
Title :
Last Update :

Signal Used: TIC; data.ms

Signal region: 8.30 to 8.32 min; height: 12341
Noise region : 8.26 to 8.29 min; Max noise 10370.0, Min noise 9821.0

Calculations	Value
Noise Points used	10
Average noise = (sum of noise)/points	10023.0
Corrected Signal = height/Average noise	2318.0
Pk-pk noise = Max noise/Min noise	549.0
Pk-pk S/N = Corrected signal/Pk-pk noise	4.2
RMS noise = SQRT(sum(square(noise-avg noise))/points)	179.5
RMS S/N = Corrected signal/RMS noise	12.9

default.m Fri Sep 24 15:15:30 2021 SYSTEM-Y

Signal to Noise Report

Data Path : C:\gcms\1\data\LOD and LOQ Troc-Norfent System-Y\
Data File : Y3387.D
Acq On : 21 Sep 2021 15:40
Operator :
Sample : LOG-FSC-203-48-2
Misc : 0.020 ug/mL Troc-Norfentanyl
ALS Vial : 1 Sample Multiplier: 1

Integration File: events.e

Method : C:\gcms\1\methods\default.m
Title :
Last Update :

Signal Used: TIC; data.ms

Signal region: 8.30 to 8.32 min; height: 14116
Noise region : 8.22 to 8.25 min; Max noise 9524.0, Min noise 9180.0

Calculations	Value
Noise Points used	9
Average noise = (sum of noise)/points	9358.3
Corrected Signal = height/Average noise	4757.7
Pk-pk noise = Max noise/Min noise	344.0
Pk-pk S/N = Corrected signal/Pk-pk noise	13.8
RMS noise = SQRT(sum(square(noise-avg noise))/points)	101.6

RMS S/N = Corrected signal/RMS noise 46.8

default.m Fri Sep 24 15:20:15 2021 SYSTEM-Y

Signal to Noise Report

Data Path : C:\gcms\1\data\LOD and LOQ Troc-Norfent System-Y\
Data File : Y3388.D
Acq On : 21 Sep 2021 15:52
Operator :
Sample : LOG-FSC-203-48-3
Misc : 0.010 ug/mL Troc-Norfentanyl
ALS Vial : 2 Sample Multiplier: 1

Integration File: events.e

Method : C:\gcms\1\methods\default.m
Title :
Last Update :

Signal Used: TIC; data.ms

Signal region: 8.30 to 8.32 min; height: 11986
Noise region : 8.22 to 8.25 min; Max noise 9529.0, Min noise 8860.0

Calculations	Value
Noise Points used	10
Average noise = (sum of noise)/points	9140.5
Corrected Signal = height/Average noise	2845.5
Pk-pk noise = Max noise/Min noise	669.0
Pk-pk S/N = Corrected signal/Pk-pk noise	4.3
RMS noise = SQRT(sum(square(noise-avg noise))/points)	179.7
RMS S/N = Corrected signal/RMS noise	15.8

default.m Fri Sep 24 15:26:26 2021 SYSTEM-Y

Signal to Noise Report

Data Path : C:\gcms\1\data\LOD and LOQ Troc-Norfent System-Y\
Data File : Y3389.D
Acq On : 21 Sep 2021 16:04
Operator :
Sample : LOG-FSC-203-48-2
Misc : 0.020 ug/mL Troc-Norfentanyl
ALS Vial : 1 Sample Multiplier: 1

Integration File: events.e

Method : C:\gcms\1\methods\default.m
Title :
Last Update :

Signal Used: TIC; data.ms

Signal region: 8.30 to 8.32 min; height: 13673
Noise region : 8.22 to 8.25 min; Max noise 9677.0, Min noise 9356.0

Calculations	Value
Noise Points used	8
Average noise = (sum of noise)/points	9573.8
Corrected Signal = height/Average noise	4099.3
Pk-pk noise = Max noise/Min noise	321.0
Pk-pk S/N = Corrected signal/Pk-pk noise	12.8
RMS noise = SQRT(sum(square(noise-avg noise))/points)	174.2
RMS S/N = Corrected signal/RMS noise	23.5

default.m Fri Sep 24 15:28:14 2021 SYSTEM-Y

Signal to Noise Report

Data Path : C:\gcms\1\data\LOD and LOQ Troc-Norfent System-Y\
Data File : Y3390.D
Acq On : 21 Sep 2021 16:16
Operator :
Sample : LOG-FSC-203-48-3
Misc : 0.010 ug/mL Troc-Norfentanyl
ALS Vial : 2 Sample Multiplier: 1

Integration File: events.e

Method : C:\gcms\1\methods\default.m
Title :
Last Update :

Signal Used: TIC; data.ms

Signal region: 8.30 to 8.32 min; height: 12117
Noise region : 8.24 to 8.28 min; Max noise 9793.0, Min noise 9395.0

Calculations	Value
Noise Points used	9
Average noise = (sum of noise)/points	9619.1
Corrected Signal = height/Average noise	2497.9
Pk-pk noise = Max noise/Min noise	398.0
Pk-pk S/N = Corrected signal/Pk-pk noise	6.3
RMS noise = SQRT(sum(square(noise-avg noise))/points)	140.6
RMS S/N = Corrected signal/RMS noise	17.8

default.m Fri Sep 24 15:29:20 2021 SYSTEM-Y

Signal to Noise Report

Data Path : C:\gcms\1\data\LOD and LOQ Troc-Norfent System-Y\
Data File : Y3392.D
Acq On : 22 Sep 2021 9:29
Operator :
Sample : LOG-FSC-203-48-2
Misc : 0.020 ug/mL Troc-Norfentanyl
ALS Vial : 1 Sample Multiplier: 1

Integration File: events.e

Method : C:\gcms\1\methods\default.m
Title :
Last Update :

Signal Used: TIC; data.ms

Signal region: 8.30 to 8.32 min; height: 13954
Noise region : 8.25 to 8.28 min; Max noise 9276.0, Min noise 8830.0

Calculations	Value
Noise Points used	9
Average noise = (sum of noise)/points	9046.0
Corrected Signal = height/Average noise	4908.0
Pk-pk noise = Max noise/Min noise	446.0
Pk-pk S/N = Corrected signal/Pk-pk noise	11.0
RMS noise = SQRT(sum(square(noise-avg noise))/points)	181.4
RMS S/N = Corrected signal/RMS noise	27.1

default.m Fri Sep 24 15:30:30 2021 SYSTEM-Y

Signal to Noise Report

Data Path : C:\gcms\1\data\LOD and LOQ Troc-Norfent System-Y\
Data File : Y3393.D
Acq On : 22 Sep 2021 9:41
Operator :
Sample : LOG-FSC-203-48-3
Misc : 0.010 ug/mL Troc-Norfentanyl
ALS Vial : 2 Sample Multiplier: 1

Integration File: events.e

Method : C:\gcms\1\methods\default.m
Title :
Last Update :

Signal Used: TIC; data.ms

Signal region: 8.30 to 8.32 min; height: 11512
Noise region : 8.21 to 8.25 min; Max noise 8644.0, Min noise 8160.0

Calculations	Value
Noise Points used	9
Average noise = (sum of noise)/points	8426.8
Corrected Signal = height/Average noise	3085.2
Pk-pk noise = Max noise/Min noise	484.0
Pk-pk S/N = Corrected signal/Pk-pk noise	6.4
RMS noise = SQRT(sum(square(noise-avg noise))/points)	189.4
RMS S/N = Corrected signal/RMS noise	16.3

default.m Fri Sep 24 15:45:47 2021 SYSTEM-Y

Signal to Noise Report

Data Path : C:\gcms\1\data\LOD and LOQ Troc-Norfent System-Y\
Data File : Y3394.D

Acq On : 22 Sep 2021 9:53
Operator :
Sample : LOG-FSC-203-48-2
Misc : 0.020 ug/mL Troc-Norfentanyl
ALS Vial : 1 Sample Multiplier: 1

Integration File: events.e

Method : C:\gcms\1\methods\default.m
Title :
Last Update :

Signal Used: TIC; data.ms

Signal region: 8.30 to 8.32 min; height: 13742
Noise region : 8.19 to 8.23 min; Max noise 8330.0, Min noise 7978.0

Calculations	Value
Noise Points used	10
Average noise = (sum of noise)/points	8155.5
Corrected Signal = height/Average noise	5586.5
Pk-pk noise = Max noise/Min noise	352.0
Pk-pk S/N = Corrected signal/Pk-pk noise	15.9
RMS noise = SQRT(sum(square(noise-avg noise))/points)	99.5
RMS S/N = Corrected signal/RMS noise	56.2

default.m Fri Sep 24 15:47:50 2021 SYSTEM-Y

Signal to Noise Report

Data Path : C:\gcms\1\data\LOD and LOQ Troc-Norfent System-Y\
Data File : Y3395.D
Acq On : 22 Sep 2021 10:05
Operator :
Sample : LOG-FSC-203-48-3
Misc : 0.010 ug/mL Troc-Norfentanyl
ALS Vial : 2 Sample Multiplier: 1

Integration File: events.e

Method : C:\gcms\1\methods\default.m
Title :
Last Update :

Signal Used: TIC; data.ms

Signal region: 8.30 to 8.32 min; height: 11541
Noise region : 8.26 to 8.30 min; Max noise 9309.0, Min noise 8896.0

Calculations	Value
Noise Points used	9
Average noise = (sum of noise)/points	9116.2
Corrected Signal = height/Average noise	2424.8
Pk-pk noise = Max noise/Min noise	413.0
Pk-pk S/N = Corrected signal/Pk-pk noise	5.9
RMS noise = SQRT(sum(square(noise-avg noise))/points)	133.1

Calculations	Value
RMS S/N = Corrected signal/RMS noise	18.2

default.m Fri Sep 24 15:56:32 2021 SYSTEM-Y

Signal to Noise Report

Data Path : C:\gcms\1\data\LOD and LOQ Troc-Norfent System-Y\
Data File : Y3396.D
Acq On : 22 Sep 2021 10:17
Operator :
Sample : LOG-FSC-203-48-2
Misc : 0.020 ug/mL Troc-Norfentanyl
ALS Vial : 1 Sample Multiplier: 1

Integration File: events.e

Method : C:\gcms\1\methods\default.m
Title :
Last Update :

Signal Used: TIC; data.ms

Signal region: 8.30 to 8.32 min; height: 13555
Noise region : 8.25 to 8.28 min; Max noise 9129.0, Min noise 8723.0

Calculations	Value
Noise Points used	9
Average noise = (sum of noise)/points	8887.2
Corrected Signal = height/Average noise	4667.8
Pk-pk noise = Max noise/Min noise	406.0
Pk-pk S/N = Corrected signal/Pk-pk noise	11.5
RMS noise = SQRT(sum(square(noise-avg noise))/points)	120.6
RMS S/N = Corrected signal/RMS noise	38.7

default.m Fri Sep 24 15:57:18 2021 SYSTEM-Y

Signal to Noise Report

Data Path : C:\gcms\1\data\LOD and LOQ Troc-Norfent System-Y\
Data File : Y3397.D
Acq On : 22 Sep 2021 10:29
Operator :
Sample : LOG-FSC-203-48-3
Misc : 0.010 ug/mL Troc-Norfentanyl
ALS Vial : 2 Sample Multiplier: 1

Integration File: events.e

Method : C:\gcms\1\methods\default.m
Title :
Last Update :

Signal Used: TIC; data.ms

Signal region: 8.30 to 8.32 min; height: 11522
Noise region : 8.20 to 8.24 min; Max noise 8830.0, Min noise 8333.0

Calculations	Value
Noise Points used	10
Average noise = (sum of noise)/points	8510.2
Corrected Signal = height/Average noise	3011.8
Pk-pk noise = Max noise/Min noise	497.0
Pk-pk S/N = Corrected signal/Pk-pk noise	6.1
RMS noise = SQRT(sum(square(noise-avg noise))/points)	140.2
RMS S/N = Corrected signal/RMS noise	21.5

default.m Fri Sep 24 15:59:11 2021 SYSTEM-Y

Signal to Noise Report

Data Path : C:\gcms\1\data\LOD and LOQ Troc-Norfent System-Y\
Data File : Y3398.D
Acq On : 22 Sep 2021 10:42
Operator :
Sample : LOG-FSC-203-48-2
Misc : 0.020 ug/mL Troc-Norfentanyl
ALS Vial : 1 Sample Multiplier: 1

Integration File: events.e

Method : C:\gcms\1\methods\default.m
Title :
Last Update :

Signal Used: TIC; data.ms

Signal region: 8.30 to 8.32 min; height: 13470
Noise region : 8.20 to 8.24 min; Max noise 8626.0, Min noise 8264.0

Calculations	Value
Noise Points used	9
Average noise = (sum of noise)/points	8426.7
Corrected Signal = height/Average noise	5043.3
Pk-pk noise = Max noise/Min noise	362.0
Pk-pk S/N = Corrected signal/Pk-pk noise	13.9
RMS noise = SQRT(sum(square(noise-avg noise))/points)	122.9
RMS S/N = Corrected signal/RMS noise	41.0

default.m Fri Sep 24 16:00:07 2021 SYSTEM-Y

Signal to Noise Report

Data Path : C:\gcms\1\data\LOD and LOQ Troc-Norfent System-Y\
Data File : Y3399.D
Acq On : 22 Sep 2021 10:54
Operator :
Sample : LOG-FSC-203-48-3
Misc : 0.010 ug/mL Troc-Norfentanyl
ALS Vial : 2 Sample Multiplier: 1

Integration File: events.e

Method : C:\gcms\1\methods\default.m
Title :
Last Update :

Signal Used: TIC; data.ms

Signal region: 8.30 to 8.32 min; height: 11369
Noise region : 8.24 to 8.26 min; Max noise 9106.0, Min noise 8750.0

Calculations	Value
Noise Points used	7
Average noise = (sum of noise)/points	8915.6
Corrected Signal = height/Average noise	2453.4
Pk-pk noise = Max noise/Min noise	356.0
Pk-pk S/N = Corrected signal/Pk-pk noise	6.9
RMS noise = SQRT(sum(square(noise-avg noise))/points)	128.2
RMS S/N = Corrected signal/RMS noise	19.1

default.m Fri Sep 24 16:00:50 2021 SYSTEM-Y

Signal to Noise Report

Data Path : C:\gcms\1\data\LOD and LOQ Troc-Norfent System-Y\
Data File : Y3400.D
Acq On : 22 Sep 2021 11:06
Operator :
Sample : LOG-FSC-203-48-2
Misc : 0.020 ug/mL Troc-Norfentanyl
ALS Vial : 1 Sample Multiplier: 1

Integration File: events.e

Method : C:\gcms\1\methods\default.m
Title :
Last Update :

Signal Used: TIC; data.ms

Signal region: 8.30 to 8.32 min; height: 13663
Noise region : 8.24 to 8.27 min; Max noise 9010.0, Min noise 8635.0

Calculations	Value
Noise Points used	8
Average noise = (sum of noise)/points	8806.6
Corrected Signal = height/Average noise	4856.4
Pk-pk noise = Max noise/Min noise	375.0
Pk-pk S/N = Corrected signal/Pk-pk noise	13.0
RMS noise = SQRT(sum(square(noise-avg noise))/points)	126.8
RMS S/N = Corrected signal/RMS noise	38.3

default.m Fri Sep 24 16:01:40 2021 SYSTEM-Y

Signal to Noise Report

Data Path : C:\gcms\1\data\LOD and LOQ Troc-Norfent System-Y\
Data File : Y3401.D

Acq On : 22 Sep 2021 11:18
Operator :
Sample : LOG-FSC-203-48-3
Misc : 0.010 ug/mL Troc-Norfentanyl
ALS Vial : 2 Sample Multiplier: 1

Integration File: events.e

Method : C:\gcms\1\methods\default.m
Title :
Last Update :

Signal Used: TIC; data.ms

Signal region: 8.30 to 8.32 min; height: 11938
Noise region : 8.25 to 8.28 min; Max noise 9348.0, Min noise 8930.0

Calculations	Value
Noise Points used	9
Average noise = (sum of noise)/points	9150.1
Corrected Signal = height/Average noise	2787.9
Pk-pk noise = Max noise/Min noise	418.0
Pk-pk S/N = Corrected signal/Pk-pk noise	6.7
RMS noise = SQRT(sum(square(noise-avg noise))/points)	123.8
RMS S/N = Corrected signal/RMS noise	22.5

default.m Fri Sep 24 16:03:13 2021 SYSTEM-Y

Signal to Noise Report

Data Path : C:\gcms\1\data\LOD and LOQ Troc-Norfent System-Y\
Data File : Y3403.D
Acq On : 24 Sep 2021 9:36
Operator :
Sample : LOG-FSC-203-48-2
Misc : 0.020 ug/mL Troc-Norfentanyl
ALS Vial : 1 Sample Multiplier: 1

Integration File: events.e

Method : C:\gcms\1\methods\default.m
Title :
Last Update :

Signal Used: TIC; data.ms

Signal region: 8.30 to 8.32 min; height: 14456
Noise region : 8.25 to 8.27 min; Max noise 9653.0, Min noise 9308.0

Calculations	Value
Noise Points used	7
Average noise = (sum of noise)/points	9477.4
Corrected Signal = height/Average noise	4978.6
Pk-pk noise = Max noise/Min noise	345.0
Pk-pk S/N = Corrected signal/Pk-pk noise	14.4
RMS noise = SQRT(sum(square(noise-avg noise))/points)	141.4

RMS S/N = Corrected signal/RMS noise 35.2

default.m Fri Sep 24 16:04:05 2021 SYSTEM-Y

Signal to Noise Report

Data Path : C:\gcms\1\data\LOD and LOQ Troc-Norfent System-Y\
Data File : Y3404.D
Acq On : 24 Sep 2021 9:49
Operator :
Sample : LOG-FSC-203-48-3
Misc : 0.010 ug/mL Troc-Norfentanyl
ALS Vial : 2 Sample Multiplier: 1

Integration File: events.e

Method : C:\gcms\1\methods\default.m
Title :
Last Update :

Signal Used: TIC; data.ms

Signal region: 8.30 to 8.32 min; height: 11992
Noise region : 8.25 to 8.28 min; Max noise 9763.0, Min noise 9370.0

Calculations	Value
Noise Points used	8
Average noise = (sum of noise)/points	9573.3
Corrected Signal = height/Average noise	2418.8
Pk-pk noise = Max noise/Min noise	393.0
Pk-pk S/N = Corrected signal/Pk-pk noise	6.2
RMS noise = SQRT(sum(square(noise-avg noise))/points)	125.0
RMS S/N = Corrected signal/RMS noise	19.4

default.m Fri Sep 24 16:05:18 2021 SYSTEM-Y

Signal to Noise Report

Data Path : C:\gcms\1\data\LOD and LOQ Troc-Norfent System-Y\
Data File : Y3405.D
Acq On : 24 Sep 2021 10:01
Operator :
Sample : LOG-FSC-203-48-2
Misc : 0.020 ug/mL Troc-Norfentanyl
ALS Vial : 1 Sample Multiplier: 1

Integration File: events.e

Method : C:\gcms\1\methods\default.m
Title :
Last Update :

Signal Used: TIC; data.ms

Signal region: 8.30 to 8.32 min; height: 13682
Noise region : 8.25 to 8.27 min; Max noise 9038.0, Min noise 8737.0

Calculations	Value
Noise Points used	6
Average noise = (sum of noise)/points	8856.2
Corrected Signal = height/Average noise	4825.8
Pk-pk noise = Max noise/Min noise	301.0
Pk-pk S/N = Corrected signal/Pk-pk noise	16.0
RMS noise = SQRT(sum(square(noise-avg noise))/points)	92.8
RMS S/N = Corrected signal/RMS noise	52.0

default.m Fri Sep 24 16:06:04 2021 SYSTEM-Y

Signal to Noise Report

Data Path : C:\gcms\1\data\LOD and LOQ Troc-Norfent System-Y\
Data File : Y3406.D
Acq On : 24 Sep 2021 10:13
Operator :
Sample : LOG-FSC-203-48-3
Misc : 0.010 ug/mL Troc-Norfentanyl
ALS Vial : 2 Sample Multiplier: 1

Integration File: events.e

Method : C:\gcms\1\methods\default.m
Title :
Last Update :

Signal Used: TIC; data.ms

Signal region: 8.30 to 8.32 min; height: 12162
Noise region : 8.24 to 8.26 min; Max noise 9360.0, Min noise 8869.0

Calculations	Value
Noise Points used	5
Average noise = (sum of noise)/points	9156.8
Corrected Signal = height/Average noise	3005.2
Pk-pk noise = Max noise/Min noise	491.0
Pk-pk S/N = Corrected signal/Pk-pk noise	6.1
RMS noise = SQRT(sum(square(noise-avg noise))/points)	165.5
RMS S/N = Corrected signal/RMS noise	18.2

default.m Fri Sep 24 16:07:14 2021 SYSTEM-Y

Signal to Noise Report

Data Path : C:\gcms\1\data\LOD and LOQ Troc-Norfent System-Y\
Data File : Y3407.D
Acq On : 24 Sep 2021 10:25
Operator :
Sample : LOG-FSC-203-48-2
Misc : 0.020 ug/mL Troc-Norfentanyl
ALS Vial : 1 Sample Multiplier: 1

Integration File: events.e

Method : C:\gcms\1\methods\default.m
Title :
Last Update :

Signal Used: TIC; data.ms

Signal region: 8.30 to 8.32 min; height: 13809
Noise region : 8.25 to 8.28 min; Max noise 9113.0, Min noise 8812.0

Calculations	Value
Noise Points used	7
Average noise = (sum of noise)/points	8968.4
Corrected Signal = height/Average noise	4840.6
Pk-pk noise = Max noise/Min noise	301.0
Pk-pk S/N = Corrected signal/Pk-pk noise	16.1
RMS noise = SQRT(sum(square(noise-avg noise))/points)	115.5
RMS S/N = Corrected signal/RMS noise	41.9

default.m Fri Sep 24 16:08:00 2021 SYSTEM-Y

Signal to Noise Report

Data Path : C:\gcms\1\data\LOD and LOQ Troc-Norfent System-Y\
Data File : Y3408.D
Acq On : 24 Sep 2021 10:37
Operator :
Sample : LOG-FSC-203-48-3
Misc : 0.010 ug/mL Troc-Norfentanyl
ALS Vial : 2 Sample Multiplier: 1

Integration File: events.e

Method : C:\gcms\1\methods\default.m
Title :
Last Update :

Signal Used: TIC; data.ms

Signal region: 8.30 to 8.32 min; height: 11961
Noise region : 8.23 to 8.25 min; Max noise 9065.0, Min noise 8523.0

Calculations	Value
Noise Points used	8
Average noise = (sum of noise)/points	8865.4
Corrected Signal = height/Average noise	3095.6
Pk-pk noise = Max noise/Min noise	542.0
Pk-pk S/N = Corrected signal/Pk-pk noise	5.7
RMS noise = SQRT(sum(square(noise-avg noise))/points)	183.3
RMS S/N = Corrected signal/RMS noise	16.9

default.m Fri Sep 24 16:09:06 2021 SYSTEM-Y

Signal to Noise Report

Data Path : C:\gcms\1\data\LOD and LOQ Troc-Norfent System-Y\
Data File : Y3409.D

Acq On : 24 Sep 2021 10:49
Operator :
Sample : LOG-FSC-203-48-2
Misc : 0.020 ug/mL Troc-Norfentanyl
ALS Vial : 1 Sample Multiplier: 1

Integration File: events.e

Method : C:\gcms\1\methods\default.m
Title :
Last Update :

Signal Used: TIC; data.ms

Signal region: 8.30 to 8.32 min; height: 13651
Noise region : 8.21 to 8.23 min; Max noise 8531.0, Min noise 8160.0

Calculations	Value
Noise Points used	6
Average noise = (sum of noise)/points	8361.5
Corrected Signal = height/Average noise	5289.5
Pk-pk noise = Max noise/Min noise	371.0
Pk-pk S/N = Corrected signal/Pk-pk noise	14.3
RMS noise = SQRT(sum(square(noise-avg noise))/points)	113.9
RMS S/N = Corrected signal/RMS noise	46.4

default.m Fri Sep 24 16:12:11 2021 SYSTEM-Y

Signal to Noise Report

Data Path : C:\gcms\1\data\LOD and LOQ Troc-Norfent System-Y\
Data File : Y3410.D
Acq On : 24 Sep 2021 11:01
Operator :
Sample : LOG-FSC-203-48-3
Misc : 0.010 ug/mL Troc-Norfentanyl
ALS Vial : 2 Sample Multiplier: 1

Integration File: events.e

Method : C:\gcms\1\methods\default.m
Title :
Last Update :

Signal Used: TIC; data.ms

Signal region: 8.30 to 8.32 min; height: 11808
Noise region : 8.25 to 8.28 min; Max noise 9303.0, Min noise 8761.0

Calculations	Value
Noise Points used	7
Average noise = (sum of noise)/points	9028.9
Corrected Signal = height/Average noise	2779.1
Pk-pk noise = Max noise/Min noise	542.0
Pk-pk S/N = Corrected signal/Pk-pk noise	5.1
RMS noise = SQRT(sum(square(noise-avg noise))/points)	167.8

RMS S/N = Corrected signal/RMS noise	16.6

default.m Fri Sep 24 16:12:56 2021 SYSTEM-Y

Signal to Noise Report

Data Path : C:\gcms\1\data\LOD and LOQ Troc-Norfent System-Y\
Data File : Y3411.D
Acq On : 24 Sep 2021 11:13
Operator :
Sample : LOG-FSC-203-48-2
Misc : 0.020 ug/mL Troc-Norfentanyl
ALS Vial : 1 Sample Multiplier: 1

Integration File: events.e

Method : C:\gcms\1\methods\default.m
Title :
Last Update :

Signal Used: TIC; data.ms

Signal region: 8.30 to 8.32 min; height: 12771
Noise region : 8.22 to 8.24 min; Max noise 8603.0, Min noise 8315.0

Calculations	Value
Noise Points used	6
Average noise = (sum of noise)/points	8482.2
Corrected Signal = height/Average noise	4288.8
Pk-pk noise = Max noise/Min noise	288.0
Pk-pk S/N = Corrected signal/Pk-pk noise	14.9
RMS noise = SQRT(sum(square(noise-avg noise))/points)	147.0
RMS S/N = Corrected signal/RMS noise	29.2

default.m Fri Sep 24 16:14:55 2021 SYSTEM-Y

Signal to Noise Report

Data Path : C:\gcms\1\data\LOD and LOQ Troc-Norfent System-Y\
Data File : Y3412.D
Acq On : 24 Sep 2021 11:26
Operator :
Sample : LOG-FSC-203-48-3
Misc : 0.010 ug/mL Troc-Norfentanyl
ALS Vial : 2 Sample Multiplier: 1

Integration File: events.e

Method : C:\gcms\1\methods\default.m
Title :
Last Update :

Signal Used: TIC; data.ms

Signal region: 8.30 to 8.32 min; height: 12034
Noise region : 8.26 to 8.29 min; Max noise 9429.0, Min noise 9014.0

Calculations	Value
Noise Points used	9
Average noise = (sum of noise)/points	9216.4
Corrected Signal = height/Average noise	2817.6
Pk-pk noise = Max noise/Min noise	415.0
Pk-pk S/N = Corrected signal/Pk-pk noise	6.8
RMS noise = SQRT(sum(square(noise-avg noise))/points)	133.2
RMS S/N = Corrected signal/RMS noise	21.2

default.m Fri Sep 24 16:15:39 2021 SYSTEM-Y
